# Photoisomerization of Heptamethine Cyanine Dyes Results in Red-Emissive Species: Implications for Near-IR, Single-Molecule, and Super-Resolution Fluorescence Spectroscopy and Imaging

**DOI:** 10.1021/acs.jpcb.2c08016

**Published:** 2023-04-03

**Authors:** Elin Sandberg, Joachim Piguet, Uliana Kostiv, Glib Baryshnikov, Haichun Liu, Jerker Widengren

**Affiliations:** † Experimental Biomolecular Physics, Dept. Applied Physics, Royal Institute of Technology (KTH), Albanova Univ Center, 106 91 Stockholm, Sweden; ‡ Dept. Science and Technology, Linköping University, Campus Norrköping, 601 74 Norrköping, Sweden

## Abstract

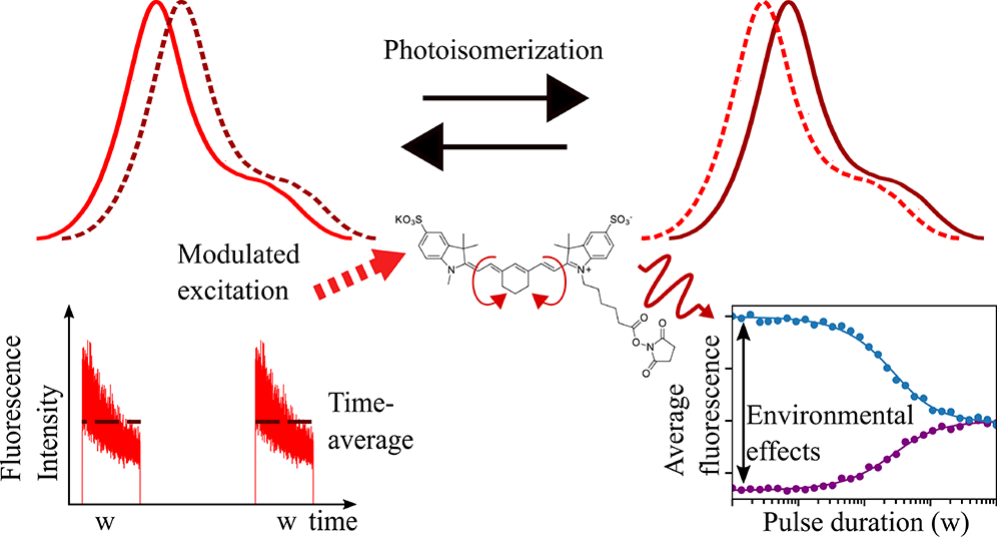

Photoisomerization kinetics of the near-infrared (NIR) fluorophore Sulfo-Cyanine7 (SCy7) was studied by a combination of fluorescence correlation spectroscopy (FCS) and transient state (TRAST) excitation modulation spectroscopy. A photoisomerized state with redshifted emission was identified, with kinetics consistent with a three-state photoisomerization model. Combining TRAST excitation modulation with spectrofluorimetry (spectral-TRAST) further confirmed an excitation-induced redshift in the emission spectrum of SCy7. We show how this red-emissive photoisomerized state contributes to the blinking kinetics in different emission bands of NIR cyanine dyes, and how it can influence single-molecule, super-resolution, as well as Förster resonance energy transfer (FRET) and multicolor readouts. Since this state can also be populated at moderate excitation intensities, it can also more broadly influence fluorescence readouts, also readouts not relying on high excitation conditions. However, this additional red-emissive state and its photodynamics, as identified and characterized in this work, can also be used as a strategy to push the emission of NIR cyanine dyes further into the NIR and to enhance photosensitization of nanoparticles with absorption spectra further into the NIR. Finally, we show that the photoisomerization kinetics of SCy7 and the formation of its redshifted photoisomer depend strongly on local environmental conditions, such as viscosity, polarity, and steric constraints, which suggests the use of SCy7 and other NIR cyanine dyes as environmental sensors. Such environmental information can be monitored by TRAST, in the NIR, with low autofluorescence and scattering conditions and on a broad range of samples and experimental conditions.

## Introduction

Fluorescence imaging in the near-infrared (NIR, 700–1700 nm) is receiving increasing interest in life science, offering major merits, including lower phototoxicity, reduced light scattering and autofluorescence, deeper imaging depths, and an extended range of emission bands for multiplexing.^1−3^ A major part of currently used NIR dyes belong to the cyanine dye category. Over the years, cyanine dyes have found extensive use not only in life science, but also as photosensitizers, mode-locking compounds and in solar cells.^4^ Motivated by this manifold of applications, the photophysics of cyanine dyes has been extensively studied.^5^ By techniques, such as transient absorption spectroscopy,^6−14^ laser-induced optoacoustic,^15,16^ photostationary absorption and fluorescence experiments,^14,17−22^ and theoretical calculations of electronic state energies and oscillator strengths,^23,24^ the main features of cyanine dyes have been well established. In room-temperature solutions, most ground-state cyanine dyes are in an all-*trans* (*N*) conformation. Following excitation into the excited singlet state, photoisomerization typically takes place with a high quantum yield (Φ_iso_), while the fluorescence quantum yield (Φ_f_) is relatively low compared to other fluorophore labels. Intersystem crossing to a triplet state typically competes with photoisomerization and takes place with a low quantum yield (Φ_isc_), which often can be disregarded. The photoisomerization is highly dependent on the local environment and is reduced by increased viscosity,^6^ increased viscous drag by head group substituents,^25^ and sterical constraints upon binding to, for example, proteins.^26^ However, despite extensive research, many photophysical mechanisms of cyanine dyes remain elusive. Particularly, NIR heptamethine dyes offer challenges, in that their larger molecular size allows an increased number of electronic and conformational state transitions to take place than in penta- or trimethine cyanine dyes emitting in the visible. Along this line, heptamethine cyanines have been found to display considerably lower Φ_iso_ than corresponding penta- and trimethine cyanine dyes, which could be attributed to higher nonradiative and solvent-mediated de-excitation rates.^27^ A corresponding added complexity also follows in the efforts to calculate and simulate the transitions of these dyes.

In life science, the use of cyanine dyes has in the last decades been further boosted by the strong development of fluorescence-based single-molecule spectroscopy (SMS) and super-resolution microscopy (SRM).^28^ Here, properties such as a large excitation cross section, high fluorescence brightness and photostability, and spectral compatibility to common laser excitation sources and single-photon counting detectors have made cyanine dyes a major fluorophore category of choice. Moreover, fluorophore blinking, caused by reversible transitions into long-lived, nonfluorescent dark states, such as triplet, photoionized and photoisomerized states, are also of central importance. At typical excitation conditions useful for SMS,^29^ such transitions can compromise molecular brightness and signal-to-background conditions, lead to precursor states of permanent photobleaching, and obscure observations of single-molecule dynamic events of interest occurring at similar timescales. At the same time, however, blinking or switching of fluorescence emitters on and off is also an absolute prerequisite for all forms of SRM.^30^ Particularly, in single-molecule localization microscopy (SMLM) SRM,^31^ cyanine dyes are the overall fluorophores of choice because of their switching properties. Yet, several questions remain open regarding underlying switching/blinking mechanisms in cyanine fluorophores^32,33^ and on how to optimize these for SRM applications (see ref (34) for a review). Here, the fact that cyanine fluorophores, such as pentamethine cyanine Cy5, spend as much as 50% of their time in a photoisomerized state under relevant excitation conditions for SMS and SRM^35^ can lead to secondary effects on other blinking mechanisms. It was recently shown that Förster resonance energy transfer (FRET) between closely spaced (<10 nm) Cy5 fluorophores in an all-trans (N) and photoisomerized cis (P) state can result in accelerated blinking, lowering the localization probability of fluorophores in SMLM.^36^ Moreover, while almost all SMS and SRM applications today are based on fluorophores emitting in the visible, there is also an interest to expand SMS and SRM techniques into the NIR to take advantage of the benefits that follow with NIR excitation/detection. This adds further motivation to explore the photophysical properties of NIR cyanine dyes in the context of SMS and SRM, as well as within fluorescence imaging in general.

In this work, we studied the photodynamics of the heptamethine NIR cyanine dye, Sulfo-Cy7 (SCy7), by transient state (TRAST) spectroscopy,^37−42^ together with fluorescence correlation spectroscopy (FCS). In TRAST, reversible transitions of long-lived dark states of fluorescent molecules can be characterized from how the time-averaged fluorescence intensity from the fluorophores varies with the modulation of the laser excitation intensity. TRAST bears similarities to FCS, in that it combines a high detection sensitivity offered by the fluorescence signal with a high environmental sensitivity acquired via the kinetics of long-lived dark transient states. However, in contrast to FCS, TRAST does not rely on single-molecule detection conditions or a high time resolution and can therefore be applied on a broader range of samples. In TRAST experiments, observing how the average fluorescence within rectangular excitation pulses varies with the duration of such pulses, a typical finding is that the fluorescence is reduced with increasing pulse durations due to a build-up of dark transient states. In FCS measurements, such dark-state relaxations are typically manifested similarly as decreases in the recorded FCS curves with increasing correlation times and with the decay amplitudes corresponding to the population probabilities of the dark states.^35,43,44^ In the TRAST measurements in this work, however, we also observed an increase in the fluorescence intensity with longer excitation durations, suggesting that SCy7 can be converted into another fluorescent state upon excitation. This increase was not observed at blueshifted excitation wavelengths or in higher, more aprotic alcohols with lower polarity. By complementary fluorescence lifetime, FCS, spectrofluorometer, and spectrophotometer measurements, we conclude that these TRAST observations can be attributed to the formation of an additional red-emissive photoisomerized state. This formation, consistent with a two-step photoisomerization process, can also be found in other NIR cyanine dyes, and at the end, we discuss effects that need to be considered in SMS and SRM as well as in NIR fluorescence measurements in general. Finally, we show that the photoisomerization and emissive state formation of SCy7 strongly depend on local viscosity, polarity, and sterical constraints. It is shown how these effects can be followed in a facile manner by TRAST, opening for additional means to monitor local microenvironments in solutions and membranes.

## Methods and Materials

### Sample Preparation

Stock solutions of Sulfo-Cyanine7 NHS ester (SSCy7) and Cyanine7 amine (amino-Cy7) (Lumiprobe GmbH, Hannover, Germany) were prepared in DMF and stored at −20 °C and then diluted in different solvents just before measurements to a final concentration of 1 μM, if not stated otherwise.

Small unilamellar vesicles (SUVs) were prepared from POPC (1-palmitoyl-2-oleoyl-*glycero*-3-phosphocholine), POPG (1-palmitoyl-2-oleoyl-*sn*-glycero-3-phosphoglycerol), and POPE (1-palmitoyl-2-oleoyl-*sn*-glycero-3-phosphoethanolamine), all from Avanti Polar Lipids. POPE-Sulfo-Cyanine7 was synthesized from POPE and SCy7. POPE (60 μL, 34.82 mM), Sulfo-Cyanine7 NHS (10 μL, 10 mM), and triethylamine (20 μL) in chloroform (1.84 mL) were stirred for 2 h in darkness at room temperature. To finish the reaction, ethylamine (10 μL) was added, and the mixture was stirred for another 10 min. The mixture was then purified by silica gel chromatography (7:3:0.4 vol/vol chloroform/methanol:water). The fraction of POPE-Sulfo-Cyanine7 was evaporated under a flow of N_2_ and dispersed in chloroform.

POPC lipid chloroform solution (1 μM), containing a fraction of POPE-Sulfo-Cyanine7 lipids of 1:500, was dried under a flow of N_2_ in a glass vial. To make SUVs, water (0.7 mL) was added to the dried lipids, vortexed, and sonicated for 4 min at 50% duty cycle (0.50 s on/off), 50% power (125 W) by a Branson SFX250 sonicator, and a 1/8″ microtip (Emerson Electric Co, St. Louis, MO, US). To remove aggregates, the SUV solution was centrifuged for 15 min at 14,000*g*, and the supernatant was filtered through a 0.2 μm spin filter (Corning, NY, USA).

All other chemicals were purchased from Sigma-Aldrich (St Louis, MO, USA).

### TRAST Experiments

In TRAST measurements, fluorophore blinking kinetics are determined by recording the average fluorescence intensity from an ensemble of fluorophores subject to modulated excitation. With the excitation modulation systematically varied on the time scales of the fluorophore dark-state kinetics, rapid blinking kinetics can be quantified without the need for time-resolved detection.^37−42^

TRAST measurements were carried out on a home-built TRAST setup based on an inverted epi-fluorescence microscope (Olympus, IX70) and modified from a previously described arrangement.^37^ In short, fluorescence is excited by a beam of a diode laser (638 nm Cobolt, 06-MLD, 200 mW, Cobolt, 06-MLD, 730 nm, 100 mW, or 785 nm 06-MLD, 250 mW) passing appropriate excitation filters (Semrock BrightLine 637/7, Chroma ET740/40x). The 638 nm and 785 nm laser beams were modulated by acousto-optic modulators (AOM; AA Opto Electronics, MQ180-A0,25-VIS and MT110-A1-IR), while the 730 nm laser was directly modulated by external triggering. The expanded laser beam was defocused by a convex lens, reflected by a dichroic mirror (ZT640rdc, Chroma T800lpxr-xt-UF2 Croma or ZT405/473/559/635/748rpc-UF3, Chroma), and then focused close to the back aperture of the objective (Olympus, UPLSAPO 60x/1.20 W) to produce a wide-field illumination in the sample (beam waist ω_0_ = 10–25 μm (1/e^2^ radius)). The fluorescence signal was collected by the same objective, passed through the same dichroic mirror and an emission filter (HQ720/150, Chroma, 809/81 Brightline Semrock 835/70 Brightline, Semrock or ZET785nf, Chroma) to remove scattered laser light and select a specific emission band of the emission spectrum, and then fed to a sCMOS camera (Hamamatsu ORCA-Flash4.0 V3). The experiments were controlled and synchronized by custom software implemented in Matlab. A digital I/O card (PCI-6602, National Instruments) was used to trigger the camera and generate random excitation pulse trains sent to the AOM driver unit. For the spectral experiments, the fluorescence signal was passed through an aperture (centered around the emission intensity maximum and with 23 percent of the fluorescence passing through, ensuring that only fluorescence from the center of the excitation volume is detected by the camera) passed through an emission filter (647 nm RazorEdge, Semrock ultrasteep long pass edge filter) and then focused into the multimode fiber (diameter 600 μm) of a fiber-coupled spectrometer (Kymera 193i, iDus InGaAs PDA DU490A, Andor). The obtained emission spectra were corrected for the wavelength-dependent detection efficiency of the spectrometer.

### TRAST Analysis

To calculate the recorded fluorescence intensity in the TRAST experiments, we used the reduced photophysical model for SCy7 in Figure 3A, with two emissive states, the all-*trans* state, *N*, and the double*-cis* state, *P*_2_, and with two (mono-)*cis* states of SCy7 nonluminescent, for simplicity represented as one state, *P*_1_. For a homogeneous solution sample and from the rate equations of a SCy7 fluorophore subject to a rectangular excitation pulse starting at *t* = 0 (Supporting Information Section S1, eqs S1–S8), the fluorescence signal recorded in our experimental setup can be described by
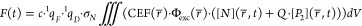
1Here, [*N*] and [*P*_2_] denote the probabilities that each of these emissive states (in either their ground or excited states) are populated in the fluorophores and *Q* = (^2^*q*_*F*_·^2^*q*_*D*_·σ_*P*_2__)/(^1^*q*_*F*_·^1^*q*_*D*_·σ_*N*_) is the relative brightness of *P*_2_ compared to *N*, where ^1^*q*_*D*_ and ^2^*q*_*D*_ denote the overall detection quantum yield of the emission from the excited singlet state of N and *P*_2_, respectively, and ^1^*q*_*F*_ and ^2^*q*_*F*_ are the fluorescence quantum yields of these states. σ_*N*_ and  denote the excitation cross section of the ground singlet state of N and P_2_, respectively,  is the collection efficiency function of the detection system, and *c* is the fluorophore concentration. Notably, differences in *Q* can thus follow from the fact that σ_*N*_ and  depend on the excitation wavelength, that ^1^*q*_*D*_ and ^2^*q*_*D*_ depend on the emission wavelength range detected, and also from differences in ^1^*q*_*F*_ and ^2^*q*_*F*_ when the dyes are in different solvents or environments.

At the onset of excitation, *F*(*t*) will show characteristic relaxation on a μs to ms time scale, reflecting changes in the population of the emissive state(*s*) (see Supporting Information Section S1, eqs S1–S8). Similar relaxations can also be observed in the time-averaged fluorescence signal resulting from a rectangular excitation pulse of duration *w*
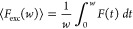
2when *w* is increased from the μs to the ms time range. Analyzing how  varies with *w* then allows the population kinetics of long-lived photoinduced states of the fluorophore to be determined, which is the general basis for TRAST monitoring.

To obtain sufficient photon counts, even for short *w*, we collected the total signal resulting from an excitation pulse train of *N* identical pulse repetitions. *N* is adjusted to maintain a constant laser illumination time, *t*_ill_ = *N*·*w*, for all *w*. The illumination time was varied between 1 and 10 ms depending on the emission count rate. A so-called TRAST curve is then produced by calculating the time-averaged fluorescence signal during excitation for each pulse train, normalized for a given pulse duration, *w*_0_
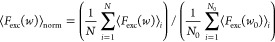
3

The pulse duration used for normalization, *w*_0_, is chosen to be short enough (typically sub-μs) not to lead to any noticeable build-up of dark transient states, yet longer than the antibunching rise time of *F*(*t*) upon onset of excitation, which typically is in the nanosecond time range.^45^

In the above expression,  represents the total signal collected from the i/th pulse in the pulse train, as defined in eq 2. By using a low excitation duty cycle, here η = 0.01–0.001, fluorophores are allowed to fully recover back to *S*_0_ before the onset of the next pulse. In the normalization step of eq 3, several parameters used to calculate *F*(*t*) in eq 1 cancel out. The final expression for  therefore becomes independent of *c* as well as of the absolute *q*_*D*_ and *q*_*F*_ values for the two emissive species.

A complete TRAST experiment consisted of a stack of 30 fluorescence images. Each image represents the total fluorescence signal from an entire excitation pulse train, captured using a camera exposure time of . Pulse durations, *w*, were distributed logarithmically between either 100 ns and 1 ms or 100 ns and 10 ms. They were measured in a randomized order to avoid bias due to time effects. An additional 10 reference frames, all using 100 ns pulse duration to avoid dark-state build-up, were inserted at regular intervals between the 30 main images to track any permanent bleaching of the sample.

The TRAST data were analyzed using a software implemented in Matlab, as previously described.^37,40,41^ The recorded TRAST data was first preprocessed by subtraction of the static ambient background, optional binning to either larger pixels or regions of interest (ROIs) within the recorded images, and correction for bleaching. The overall bleaching was maximally 5–10% of the total detected intensity.

In all measurements, TRAST curves were produced by calculating  within a ROI corresponding to a 10–25 μm radius (depending on the alignment and laser used) in the sample plane, centered on the excitation beam. Fitting of photophysical rate parameters was then performed by simulating theoretical TRAST curves using eqs 1–3 and comparing them to the experimental data. The set of rate parameter values best describing the experimental data was then found using nonlinear least-squares optimization. In the fit, the excited-state lifetime, τ_*f*_, of *N* was fixed to its fitted value determined by time-correlated single photon counting (TCSPC) measurements (see Results and Discussion) and with 1/τ_*f*_ comprising all deactivation (including isomerization) rates from the excited states of *N*. Relative differences in the excitation rates between *N* and *P*_2_ are accounted for in the relative brightness parameter, *Q* (eq 1), but could only be indirectly determined for *P*_2_ (as part of a back-isomerization cross section, see Results and Discussion). For *N*, an average singlet excitation rate, , was calculated for each ROI using eq S9 (see the Supporting Information for details) using an excitation cross section of σ_*N*_ = 9.2 × 10^–16^ cm^2^ ^46^ (at 750 nm, corrected for the excitation wavelengths and solvents used).

### FCS Experiments

FCS measurements were performed on a commercial, epi-illuminated, confocal laser scanning microscope (Olympus FV1200). Solution samples with SCy7 fluorophores, as free labels, or in SUV preparations (as described above) were excited by the focused beam of a 638 nm (338 nm, 1/e^2^ radius) or 780 nm diode laser (LDH-D-C-640 and LDH-D-C-780, both from PicoQuant GmbH, Berlin) in continuous wave. The emitted fluorescence was collected back through the microscope objective (UPlanSApo 60x/1.2w, Olympus), passed through a dichroic mirror (ZT405/488/635rpc-UF2, Chroma or T800lpxr-xt-UF2, Chroma) and an emission filter (HQ720/150, Chroma, 809/81 Brightline, Semrock, Semrock or 835/70 Brightline, Semrock), and focused onto a pinhole (50 μm diameter) in the back focal plane. The fluorescence signal was finally split and directed on two avalanche photodiodes (Tau-SPAD, PicoQuant GmbH, Berlin), whose signals were collected by a data acquisition card (Hydraharp 400, Picoquant, Berlin).

### FCS Analysis

In the FCS measurements, for freely diffusing fluorescent molecules undergoing dark-state transitions, the autocorrelation curves of the recorded fluorescence intensity, *F*(*t*), can be described by
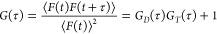
4where *G*_*D*_(τ) denotes the translational diffusion-dependent part and *G*_*T*_(τ) signifies the contribution from photoinduced dark state transitions. *G*_*D*_(τ) can be expressed as
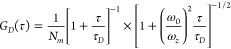
5with ω_0_ and ω_*Z*_ denoting the distances from the center of the laser beam focus in the radial and axial directions, respectively, at which the collected fluorescence intensity dropped by a factor of 1/*e*^2^ compared to its peak value. *N*_*m*_ is the mean number of fluorescent molecules within the detection volume. τ_*D*_ is the characteristic diffusion time of the fluorescent molecules, given by the diffusion coefficient *D* as .

For a fluorophore with one emissive state (within which excitation/deexcitation cycles between a ground and excited singlet state take place on a time scale much faster than the correlation times considered) and with no dark state transitions, the blinking term in eq 4, *G*_*T*_(τ) = 1. Otherwise, with *n* dark transient states and for τ much longer than the antibunching relaxation times of the fluorophores, *G*_*T*_(τ) can be expressed as a normalized set of relaxation terms,^47^ averaged over the confocal detection volume and weighted by the square of the detected molecular brightness of the molecules, 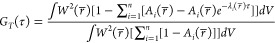
6

Here, , with σ_exc_ denoting the excitation cross section of the emissive state.  are the eigenvalues and  the related amplitudes, reflecting the population build-up of the different photoinduced nonfluorescent states. At steady state and with no photobleaching, the sum of the population probabilities for *S*_0_ and *S*_1_ together with  equals one.

For a diffusing cyanine fluorophore, which can be considered to undergo *trans*–*cis* isomerization between two states only, a fluorescent *trans* (N) state and a dark mono-*cis* (*P*_1_) state, and assuming uniform excitation conditions within the FCS detection volume, the recorded autocorrelation curves (FCS curves) can be expressed as^35,44^
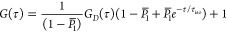
7where  denotes the averaged steady-state fraction of *P*_1_ in the detection volume upon excitation and τ_iso_ the average isomerization relaxation time. For recorded FCS curves fitted to eq 7, *S* = ω_z_/ω_0_ was fixed to 6.8 as determined from volume calibration measurements. *N*_*m*_, *A*_iso_, τ_iso_, and τ_*D*_ are the fitted parameters, with the fitting based on a nonlinear least-squares optimization routine written in Python.

For a cyanine fluorophore which apart from an all-*trans* state, *N*, can photoisomerize into a nonfluorescent mono-*cis* (*P*_1_) and an emissive double-*cis* (*P*_2_) conformation, as described by the model of Figure 3, eq 6 changes into
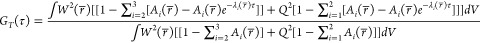
8

Here, the amplitudes  refer to the steady-state populations of *N*, *P*_1_, and *P*_2_ for *i* = 1, 2, and 3, respectively, and  are the corresponding relaxation rates/eigenvalues.  refers to the molecular brightness of *N*, and *Q* is the relative brightness of *P*_2_ compared to *N*, as defined in eq 1. With the population probabilities for *N*, *P*_1_, and *P*_2_ after the onset of constant excitation,  at time *t* = 0 and , and , defined in Supporting Information, Section S1 (eqs S1–S8), eq 8 can be written as
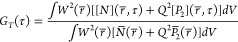
9Here,  and  are the steady-state populations of *N* and *P*_2_ (also denoted  and  above). Fitting of photophysical rate parameters was then performed by a program written in Python, simulating theoretical FCS curves using eq 9 and comparing them to the experimental data. Similar to the fitting of the experimental TRAST curves, the set of rate parameter values best describing the experimental data was then found using nonlinear least-squares optimization. In the fit, the excited-state lifetime, τ_*f*_, of *N* was fixed to the fitted value determined by TCSPC measurements (see Results and Discussion). In the fits, the ratio of *S* = ω_*z*_/ω_0_ was fixed to 6.8 and τ_*D*_ in the *G*_*D*_(τ) term was fitted as an individual parameter to the separate curves.

### Fluorescence Lifetime Measurements

TCSPC lifetime measurements were performed using the same experimental setup as for the FCS measurements but now with the excitation lasers operated in the pulsed mode. Instrument response functions were determined from the back-reflected light from the laser excitation pulses. The signals were fed into a data acquisition card (Hydraharp, Picoquant GmbH), deconvoluted, and then fit to an exponential decay based on nonlinear least-squares minimization (Symphotime, Picoquant GmbH).

### Spectrofluorometry/Spectrophotometry

Absorption and emission spectra of solution samples with SCy7 fluorophores in different solvents were measured with a spectrofluorometer/spectrophotometer (Edinburgh Instruments FS5, Mettler Toledo UV5) using different cuvette sizes (1 to 10 mm) to maximize the signal while avoiding inner filter effects. Additionally, fiber-coupled spectrometer measurements were made in the wide-field TRAST instrument, as described above.

## Results and Discussion

### FCS and TRAST Measurements of SCy7 in Aqueous Solution

**Figure 1:**
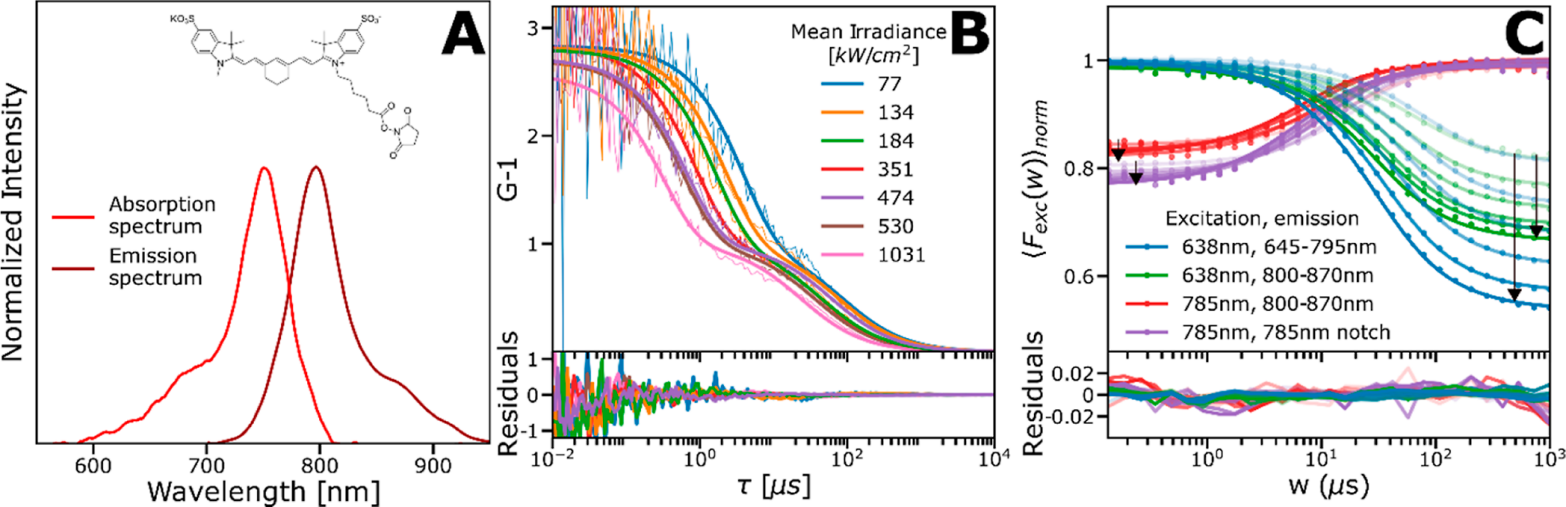
(A) Top: Structure of SCy7. Bottom: Absorption and emission spectra of SCy7. (B) FCS curves recorded from SCy7 in PBS solution (12 mM, pH 7.2), excited by a 638 nm laser. Emission filter: 645–795 nm (B-filter). Fitted curves, using a double photoisomerization model (eq 8, thick lines), fitting residuals below. See main text for further details. (C) TRAST-curves recorded from SCy7 in PBS solution (12 mM, pH 7.2) using a 638 nm (blue and green dots) or 785 nm (purple and red dots) excitation laser. Increasing color intensities in the TRAST-curves, as well as the direction of the arrows in the figure, represent increasing Φ_exc_ applied in the measurements. Applied Φ_exc_ at 638 nm excitation were [0.6, 1, 1.4, 2, 2.6, 2.9, 3.9] kW/cm^2^ and at 785 nm excitation were [1.7, 2.3, 3.3, 4.2, 4.9, 6.5] kW/cm^2^. Fitted curves, based on a double photoisomerization model (eqs 1–3, lines) with fitting residuals (below). See the main text for further details.

First, FCS measurements of SCy7 in PBS (12 mM, pH 7.2) were performed at 638 and 785 nm excitation, respectively. The structure of SCy7 and its absorption and emission spectra are shown in Figure 1A. Figure 1B shows FCS recorded at 638 nm excitation at different excitation intensities, Φ_exc_, with an emission band pass filter of 645–795 nm covering the more blue-shifted part of the emission spectrum of SCy7 (hereinafter referred to as the B-filter). The recorded FCS curves displayed dark-state relaxations with largely constant, prominent (∼70%) amplitudes, with relaxation times which decreased with higher Φ_exc_ and overall consistent with reversible excitation-driven isomerization.^35,44^ Under identical excitation conditions, switching to another emission filter with a transmission band more into the NIR (770–850 nm, hereinafter referred to as the R-filter), lower dark-state relaxation amplitudes with somewhat longer relaxation times were observed in the FCS curves (Supporting Information, Figure S1A). Recorded FCS curves with 780 nm excitation and a 800–870 nm emission filter (where the fluorescence was too low to produce reliable FCS curves with 638 nm excitation) did not show any detectable dark-state relaxation (Supporting Information, Figure S1B).

We then performed TRAST measurements on the same sample (SCy7 in PBS) at the same excitation wavelength (638 nm), with different Φ_exc_ applied. The recorded TRAST curves (blue and green points in Figure 1C) displayed dark-state relaxation processes with more than an order of magnitude slower relaxation times than in the FCS experiments (Figure 1B). Like in the FCS experiments, the relaxation times decreased with increasing Φ_exc_. Unlike the FCS experiments, however, the relaxation amplitudes were not constant but decreased with lower Φ_exc_. These observations are also consistent with reversible excitation-driven isomerization, where the much lower range of Φ_exc_ applied is the reason for the slower relaxation times compared to those in the FCS experiments. The lower relaxation amplitudes with lower Φ_exc_ observed in the TRAST curves can be attributed to non-photoinduced, thermal back-isomerization from the dark photoisomer, where for lower Φ_exc_ (<5 kW/cm^2^, 638 nm excitation), this thermal back-isomerization is no longer negligible compared to the excitation-driven back-isomerization.^35^ Similar to the observation from the FCS experiments (Figure S1A), keeping the excitation and all other experimental conditions the same, the relaxation amplitudes of the TRAST curves generally decreased when the R-filter was used versus when the B-filter was used (green versus blue data points in Figure 1C). Notably, upon excitation of the same SCy7 sample at 785 nm using two different emission filters (R-filter and 785 nm notch, hereinafter referred to as the far-red, FR-filter), the recorded TRAST curves showed an inverse relaxation (red and purple data points in Figure 1C), reflecting that the recorded fluorescence emission from SCy7 increased with time after onset of excitation. This indicates that there is an excitation-induced generation of an additional more redshifted emissive state of SCy7 and that under these conditions, the detected brightness of this photoinduced emissive state exceeds that of its all-*trans* isomer, *N*. The higher inverse amplitudes observed in the TRAST curves recorded with the FR-filter versus the R-filter (purple versus red curves in Figure 1C) further support this interpretation. At 638 nm excitation, and with similar Φ_exc_, the fact that the relaxation amplitudes in both the TRAST (Figure 1C) and FCS curves (Figure S1A) decreased with the use of more redshifted emission filters is also in agreement with an additional, excitation-induced, redshifted emissive state of SCy7. If there would be just one emissive state of SCy7 and assuming that its emission spectrum does not depend on the excitation history or excitation wavelength (Kashás rule^48^), no changes in the TRAST curves would be expected. For both 638 and 785 nm excitation, and for the different emission filters used, the overall Φ_exc_ dependence of the dark-state relaxations of SCy7 observed in both the TRAST and FCS experiments (Figure 1B,C) is consistent with reversible, largely excitation-driven transitions between at least two emissive and one nonemissive state. The overall emission filter dependence observed in the TRAST and FCS curves are further exemplified in the TRAST curves in Figure S2A–C, recorded with different excitation wavelengths applied (638, 730, and 785 nm).

### Spectral-TRAST and Fluorescence Lifetime Measurements of SCy7 in Aqueous Solution

**Figure 2:**
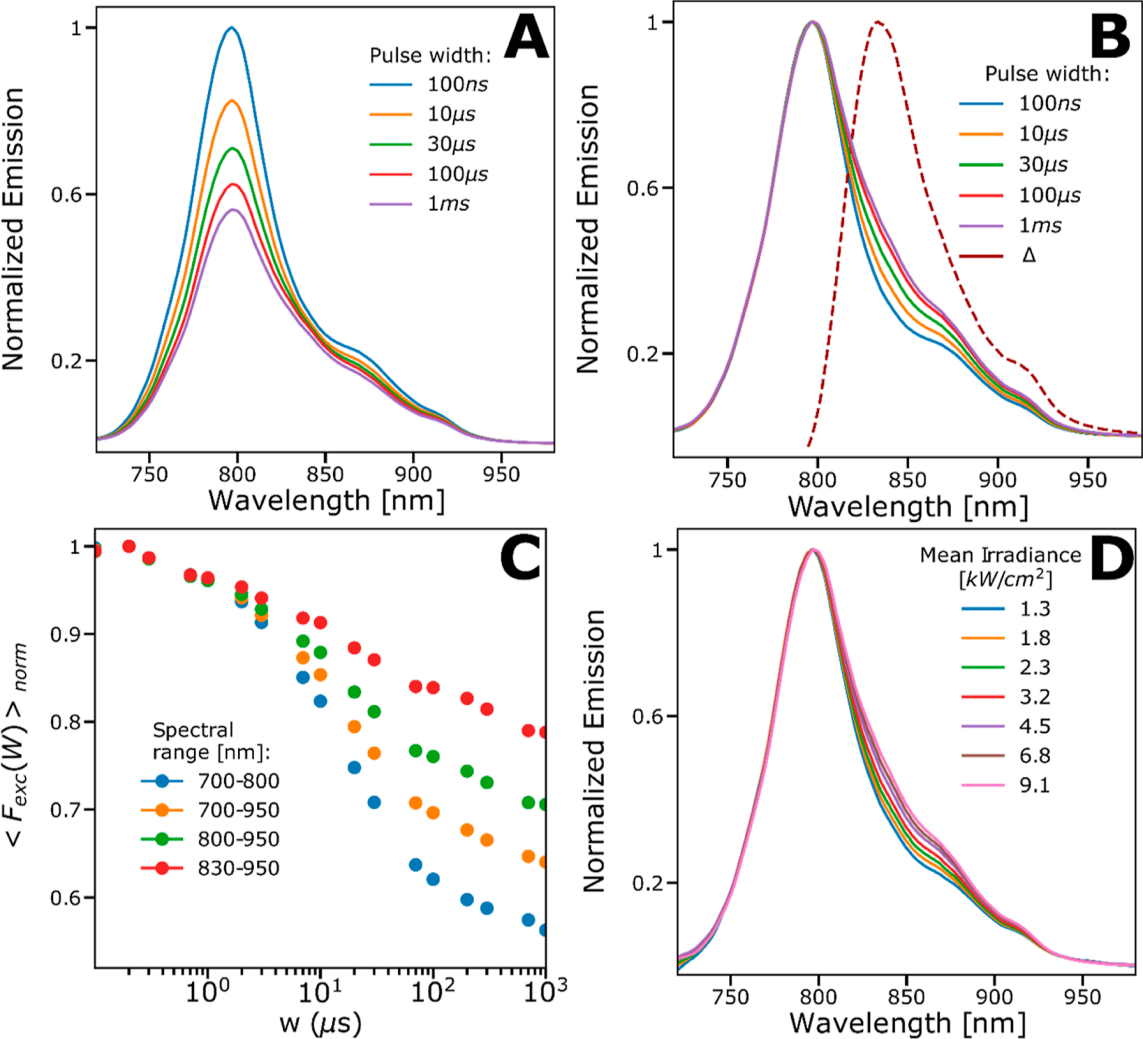
Spectral-TRAST measurements of SCy7 in PBS solution (12 mM, pH 7.2) at 638 nm excitation. The spectra in (A,B) are measured at Φ_exc_ = 3.9 kW/cm^2^ using excitation pulse trains with a constant duty cycle of 0.01 and with different pulse widths, *w*. (A) Emission spectra obtained for different w (specified in legend), normalized with the emission maximum retrieved at 100 ns. (B) Emission spectra obtained for different *w* (specified in legend), normalized with emission maximum for each curve. Dashed curve: By subtracting the normalized emission spectrum recorded with *w* = 100 ns (blue curve) from the emission spectrum recorded with *w* = 1 ms (violet curve), we obtain the approximate emission spectrum of the emissive photoisomerized species of SCy7 (dashed brown curve, Δ), generated primarily for longer *w*. (C) TRAST curves generated from fluorescence within different spectral windows of the spectra in (A) and with different spectral windows specified in the legend. The TRAST curves were normalized, so that the fluorescence intensity recorded with *w* = 100 ns within the different spectral windows [blue curve in (A)] was set to unity. (D) Emission spectrum obtained for different mean irradiances (specified in the legend) with a constant *w* = 1 ms and a constant duty cycle of 0.01. The spectra are normalized by setting their emission maxima to 1.

In addition to the excitation-driven isomerization and back-isomerization between an emissive all-*trans*, *N*, and a largely nonemissive mono-*cis, P*_1_, states typically observed for cyanine dyes in FCS experiments,^35,44^ the TRAST and FCS experiments described above thus altogether indicate that there is at least one additional isomerized state generated upon excitation, which has a redshifted emission compared to the all-*trans* isomer, *N*. To verify this hypothesis and to obtain a more continuous spectral view of how the SCy7 emission can depend on the excitation, we established so-called spectral-TRAST experiments, in which we recorded the fluorescence spectra of SCy7 upon excitation by rectangular pulse trains with different pulse durations, *w*, as regularly applied in TRAST experiments. The generation of an additional redshifted, isomerized state upon excitation, as suggested by the TRAST and FCS data presented above, should then lead to an overall redshift in these spectra with longer *w.* Results of such experiments are shown in Figure 2A–D. Indeed, at 638 nm excitation and with increased *w* (but keeping the excitation pulse train duty cycle and average intensity the same), the recorded fluorescence emission spectra (Figure 2A) displayed a prominent decrease at the emission peak wavelength (∼790 nm) and a more minor decrease at longer wavelengths. When normalizing the emission spectra to unity at the peak wavelength (Figure 2B), we thus observe a clear relative redshift in the recorded spectra with longer *w*, consistent with the hypothesis. By taking the difference between the normalized spectra in Figure 2B, with the longest and shortest excitation pulse durations applied (100 ns and 1 ms), we can also obtain the approximate, redshifted emission spectrum of the photoisomerized state (dashed line in Figure 2B). Plotting the fluorescence intensity within different spectral bands of the spectra in Figure 2B as a function of *w* (Figure 2C) then allows us to generate TRAST curves for different emission ranges over the full emission spectrum of SCy7. In the TRAST curves of Figure 2C, we observe the same trends as in the previous TRAST and FCS experiments; a clear decrease in the relaxation amplitudes for longer emission wavelengths. Increasing Φ_exc_ with constant *w* (1 ms) led to similar effects on the emission spectrum (Figure 2D), consistent with an increased relative contribution from a redshifted emissive photoisomerized state.

To gain further support for our hypothesis of an additional redshifted emissive state, we performed fluorescence lifetime measurements of SCy7 under similar but sub-ns pulsed, excitation conditions (at 638 or 780 nm) using different emission band pass filters. At 638 nm excitation and with the B-filter, a monoexponential decay with a lifetime of τ_*f*_ = 0,52 ns was observed (Figure S3A). This suggests that the emission in this case originates from one emissive state. With the same excitation, using the R-filter, the fluorescence decay was clearly better fitted as a biexponential decay (Figure S3B), with a first, fixed lifetime τ_*f*_ = 0.52 ns and a second lifetime fitted to τ_*f*2_ = 0.3 ns. A similar biexponential fluorescence decay was also observed at 780 nm excitation using the same emission filter (Figure S3C). The fluorescence decays recorded with the R-filter thus seem to originate from more than one emissive species. Additional emissive species of hepta- and pentamethine cyanines have also been reported to be generated by phototruncation. However, these light-induced species are blueshifted rather than redshifted, irreversibly formed, and not reversible and were not generated from ring-substituted cyanines, like SCy7.^49^ We also did not find any evidence of phototruncated species from SCy7 in our experiments (data not shown).

### Photophysical Model and Parametric Fitting of Recorded FCS and TRAST Curves

**Figure 3:**
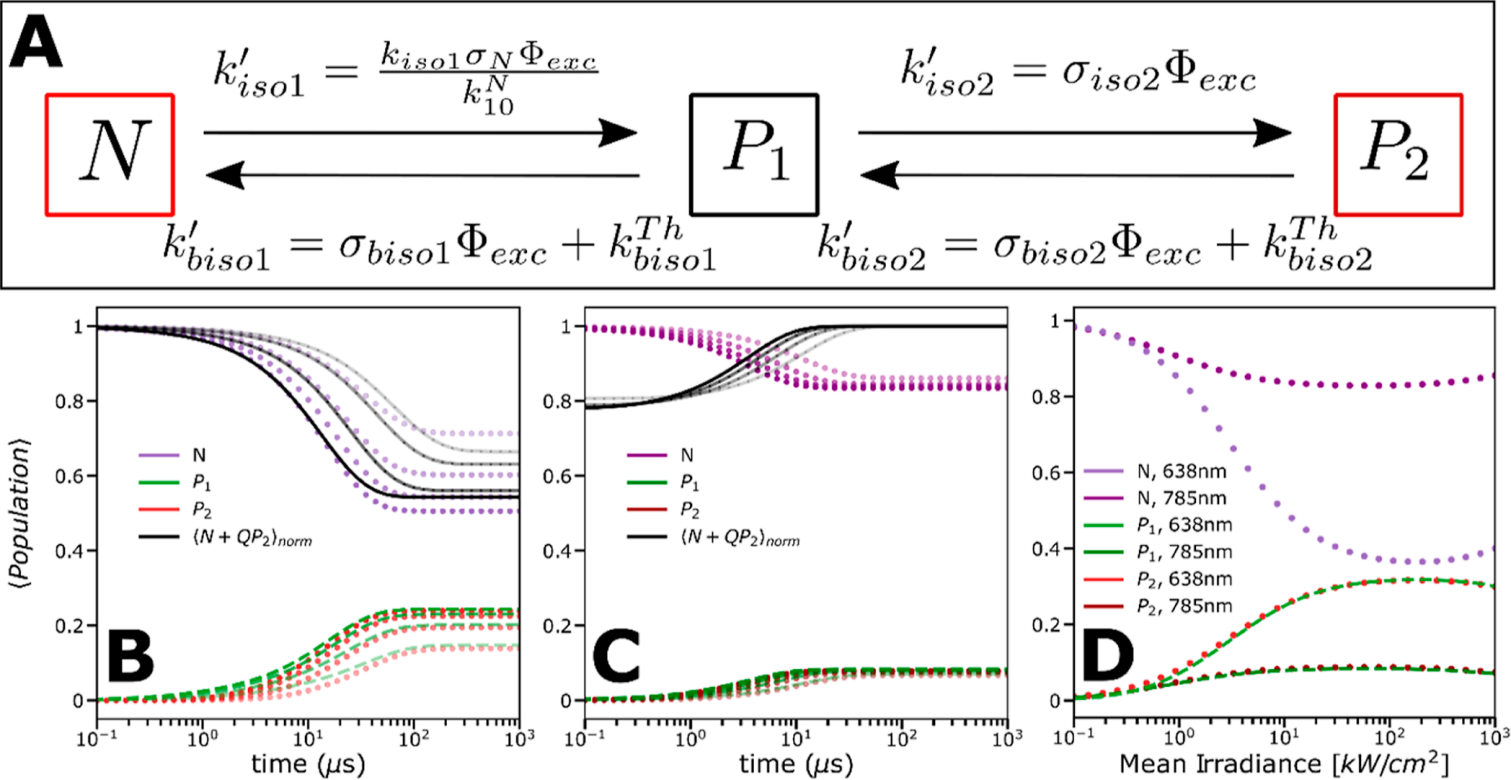
A–C. (A) 3-state isomerization model for SCy7, where *N* is the emissive all-*trans* state, *P*_1_ is a photoisomerized nonfluorescent *cis*-state, and *P*_2_ is a second photoisomerized state with redshifted emission compared to N. *k*_iso1_´, *k*_biso1_´, *k*_iso2_´,and *k*_biso2_´ are the effective isomerization rates, as described in the Supporting Information (Section S1, eqs S3 and S4); see main text for further details. (B,C) Calculated populations of *N*, *P*_1_, and *P*_2_ over time after onset of excitation at 638 nm (B) and 785 nm (C) and based on the fitted parameter values, as specified in the main text and in Table 1. The color (see legend) indicates which state is simulated, and increasing color intensities represent higher *Φ*_exc_applied. The *Φ*_exc_ values used in the calculations were [1, 2, 2.9, 3.9] kW/cm^2^ for 638 nm excitation (B) and [1.7, 3.3, 4.9, 6.5] kW/cm^2^ for 785 nm excitation (C). Black curves represent the resulting, total fluorescence from *N* and *P*_2_. (D) Calculated steady-state populations upon CW excitation based on rates and cross-sections, as obtained from fitting of the experimental TRAST curves to the model in Figure 3A, as specified in the main text and in Table 1. The steady-state populations are plotted versus Φ_exc_ for both 638 and 785 nm excitations. Colors in legend indicate which state population is calculated and for what excitation wavelength. The steady state is affected by thermal rates in the excitation irradiance range typically used in TRAST experiments (<10 kW/cm^2^), but for the irradiance range used in the FCS experiments (>70 kW/cm^2^), they have a small impact on the steady-state populations of *N*, *P*_1_, and *P*_2_.

Taken together, the FCS, TRAST, spectral-TRAST, and fluorescence lifetime measurements of SCy7 in PBS solution suggest a photophysical model for this dye, as depicted in Figure 3A. In the absence of excitation, SCy7 is in an all-*trans* state, *N*. The fluorescence spectrum for *N* corresponds to the spectral-TRAST spectrum for short *w* (≪ the relaxation time of ∼10 μs) in Figure 2A, and both this spectrum and its excitation spectrum can be expected to correspond to those reported for this dye, as obtained by regular spectrofluorometric measurements. The fluorescence lifetime of *N* corresponds to that measured with the B-filter (Figure S2A, 0,52 ns). Upon excitation, SCy7 can then not only equilibrate with a dark mono-*cis*, *P*_1_, conformation but also with an additional photoisomerized state, *P*_2_, which has redshifted excitation and emission spectra and a shorter fluorescence lifetime. In principle, SCy7 can reversely photoisomerize between multiple states, including intermediary twisted states, a double-isomerized state, and two (mono-)*cis* states. For simplicity, however, we restrict ourselves to two photoisomerized states in our model, with *P*_1_ then representing a presumably nonemissive, mono-*cis* state of SCy7 and *P*_2_ an emissive state (with redshifted emission) and shorter excited-state lifetime compared to the all-*trans N* state. The kinetic model is depicted in Figure 3A. The effective rates and the differential equations governing the population of the *N*, *P*_1_, and *P*_2_ states are described in Supporting Information, Section S1 (eqs S1–S8).

In the model, we consider state transitions occurring on a time scale much longer than the equilibration between the ground and excited singlet states within the *N*, *P*_1_, and *P*_2_ states upon onset of excitation light (their antibunching times). Given the short, sub-ns excited-state lifetimes we observe for SCy7, excited singlet-state populations will be low for the Φ_exc_ applied, particularly in the TRAST experiments. Consequently, the effective rates of intersystem crossing will be low compared to the typical triplet-state decay rates found in air-saturated aqueous solutions (∼0.5 μs^–1^).^35,43^ Moreover, intersystem crossing will also be effectively outcompeted by the higher isomerization and back-isomerization rates of the *N*, *P*_1_, and *P*_2_ states. Triplet state formation within *N*, *P*_1_, and *P*_2_ can thus be neglected. In agreement with this and apart from FCS curves recorded at the highest Φ_exc_ applied (∼MW/cm^2^), in which a minor relaxation (relative amplitude of a few percent) in the μs time range may be observed (Figure 1B), the recorded TRAST and FCS curves of SCy7 did not indicate any significate triplet-state buildup. Hence, in the model of Figure 3A, we only need to consider the effective rates of isomerization and back-isomerization, with *k*_iso1_´ = *k*_iso1_σ_*N*_·Φ_exc_/*k*_10_^*N*^ and *k*_iso2_´ = σ_iso2_·Φ_exc_ denoting the isomerization rates from *N* to *P*_1_ and from *P*_1_ to *P*_2_, respectively, and *k*_biso1_´ = σ_biso1_·Φ_exc_ + *k*_biso1_^Th^ and *k*_biso2_´ = σ_biso2_·Φ_exc_ + *k*_biso2_^Th^ signifying the back-isomerization rates from *P*_1_ to *N* and from *P*_2_ to *P*_1_, respectively. Here, *k*_iso1_ is the isomerization rate from the excited singlet state of *N* to *P*_1_ and σ_iso2_, σ_biso1_, and σ_biso2_ represent cross sections for *P*_1_-to-*P*_2_ isomerization, *P*_1_-to-*N* back-isomerization, and *P*_2_-to-*P*_1_ back-isomerization, respectively, as defined in Supporting Information, Section S1 (eqs S3 and S4). *k*_biso1_^Th^ and *k*_biso2_^Th^ denote the thermal back-isomerization rates from *P*_1_ to *N* and from *P*_2_ to *P*_1_, respectively. Here, given that most cyanine dyes are in an all-*trans* (*N*) conformation at the thermodynamic equilibrium,^4,28,35^ we neglect any thermal isomerization within SCy7 and thus assume that SCy7 fully returns to its *N* state in the absence of excitation. Finally, in the fitting of the parameter values of the model of Figure 3A to the experimental TRAST and FCS curves, the relative fluorescence brightness of *P*_2_ compared to *N*,  (see eq 1), was also included as a fitting parameter.

**Figure 4:**
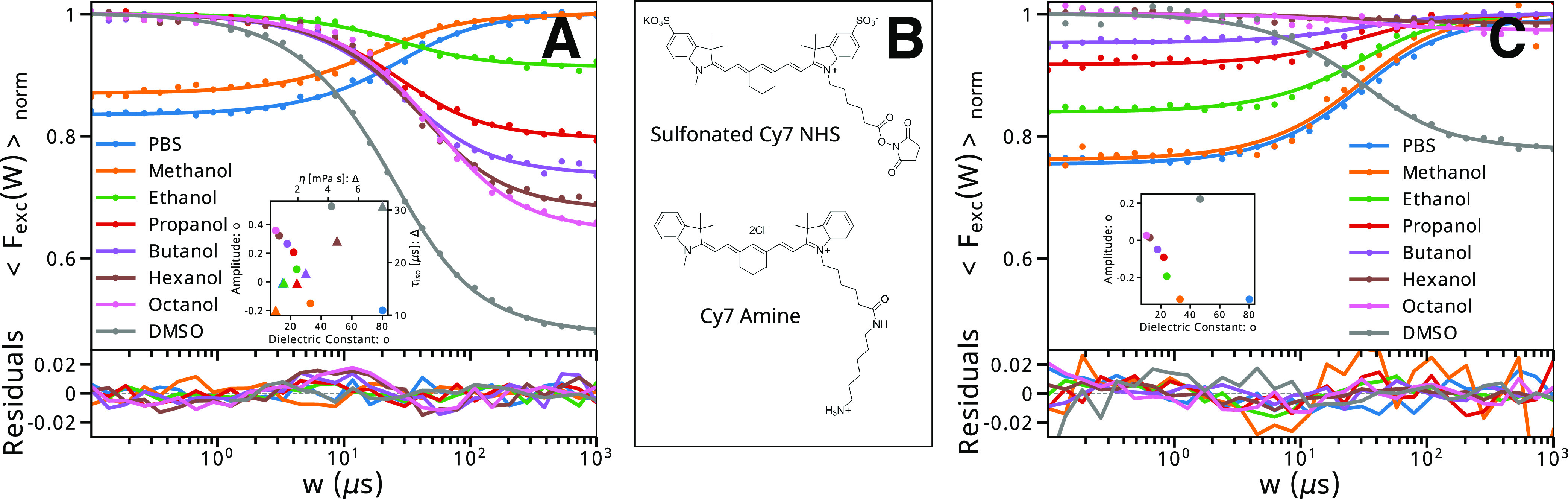
TRAST curves measured in different solvents. (A) TRAST curves recorded from SCy7 in PBS, in different alcohols, as well as in DMSO. All curves were measured at 785 nm excitation with Φ_exc_ = 1.1 kW/cm^2^ and emission detected using the R-filter. Experimental data are represented by dots. The TRAST curves were individually fitted to a one-exponential relaxation model (solid lines) for the population change in SCy7 upon onset of excitation, with the amplitude and relaxation time as the only fitted parameters. Fitting residuals plotted below. Inset: Fitted relaxation times and amplitudes plotted versus solvent viscosity and solvent polarity, respectively. See the main text for further discussion. (B) Structures of SCy7 (top) and amino-Cy7 (bottom). (C) Corresponding experimental and fitted TRAST curves as in (A), but recorded from amino-Cy7, otherwise in the same solvents and under the same experimental conditions as the curves in (A). See the main text for further discussion.

Next, with the photophysical model for SCy7 settled, we reverted to the recorded TRAST and FCS curves of SCy7 in PBS solution to fit the parameter values of the model to the recorded curves.

First, we fitted the parameter values of model to the FCS curves of SCy7 in PBS, measured at 638 nm excitation with varying Φ_exc_, and generated from fluorescence emission in the B-filter range (Figure 1B). With 638 nm excitation, the fluorescence in this emission range was found to be monoexponential (Figure S3A), and no change in the shape of the emission spectrum within this emission range was observed for different *w* (Figure 2A). This indicates that the FCS curves of Figure 1B are generated from fluorescence originating from N only. In the fit, we could thus fix *Q* to 0. Moreover, for the effective back-isomerization rates, *k*_biso1_´ and *k*_biso2_´, and at the relatively higher Φ_exc_ applied in the FCS experiments, the contribution from the thermal terms could be neglected compared to the excitation-driven terms. *k*_biso1_^Th^ and *k*_biso2_^Th^ could thus also be fixed to 0 in the fit. With these prerequisites, with the initial condition for the state populations across the FCS detection volume set according to eq S5, we then fitted the FCS curves of Figure 1B globally, as described in the Methods and Materials section, with *k*_iso_, σ_biso1_, σ_iso2_, and σ_biso2_ fitted as global parameters and with *Q*, *k*_biso1_^Th^, and *k*_biso2_^Th^ all fixed to 0. σ_*N*_ was scaled based on the absorption spectrum from SCy7, as given in Figure 1A, and the maximum extinction coefficient as stated by the manufacturer (240,600 L·mol^–1^ cm^–1^) and was then fixed to 1.56 × 10^–16^ cm² for 638 nm excitation. Moreover, *k*_10_^*N*^ = 1/τ_*f*_ – *k*_iso_, with τ_*f*_ fixed to 0.52 ns, as determined by TCSPC for *N*. The fitted curves were found to well reproduce the experimental data, with the following fitted parameter values *k*_iso_(638 nm) = 12.5 μs^–1^, σ_biso1_(638 nm) = 0.011 × 10^–16^ cm^2^, σ_iso2_(638 nm) = 0.3 × 10^–16^ cm^2^, and σ_biso2_(638 nm) = 0.13 × 10^–16^ cm^2^.

Next, we fitted the TRAST curves recorded at 638 and 785 nm excitation (Figure 1C) following the procedure described in Material and Methods. In the TRAST measurements, much lower Φ_exc_ were applied, compared to the FCS measurements. The thermal rates, *k*_biso1_^Th^ and *k*_biso2_^Th^, could then no longer be neglected compared to the excitation-driven terms of the back-isomerization rates, and *k*_biso1_^Th^ and *k*_biso2_^Th^ were thus included as global parameters in the fitting together with *k*_iso_, σ_biso1_,σ_iso2_, and σ_biso2_. First, we fitted the TRAST curves measured under 638 nm excitation, including both emission regions (B-filter and R-filter) in the same global fitting (blue and green data points in Figure 1C). Similarly, as for the FCS data, σ_*N*_ was fixed to 1.56 × 10^–16^ cm^2^ and *k*_10_^*N*^ = 1/τ_*f*_ – *k*_iso_, with τ_*f*_ fixed to 0.52 ns. For the TRAST curves recorded in the B-filter region, *Q* was fixed to 0 (for the same reasons as given above for the FCS fitting), and for the curves from the longer emission wavelength region (R-filter), *Q* was fitted globally. The fitting resulted in curves which could well reproduce the experimental TRAST curves (blue and green curves in Figure 1C) and yielded the following global parameter values: *k*_iso_(638 nm) = 14 μs^–1^, σ_biso1_(638 nm) = 0.012 × 10^–16^ cm^2^, σ_iso2_(638 nm) = 0.2 × 10^–16^ cm^2^, σ_biso2_(638 nm) = 0.2 × 10^–16^ cm^2^, *k*_th1_(638 nm) = 0.034 μs^–1^, and *k*_th2_(638 nm) = 0.011 μs^–1^. *Q* was fitted to 0.56 for the TRAST curves recorded within the R-filter region. It can be noted that the fitted cross-sections σ_biso1_, σ_iso2_, and σ_biso2_ agree well with those obtained from the FCS measurements, which is to be expected given the same excitation wavelength used. Likewise, a *Q* value of 0.56 for the longer wavelength emission data is in line with a non-negligible emission from *P*_2_ in this wavelength range. The overall dark-state population generated upon excitation is then lower, which is a reason for the lower TRAST amplitudes observed. Next, we fitted the TRAST curves recorded under 785 nm excitation (red and purple data points in Figure 1C). σ_*N*_(785 nm) was calculated based on the absorption spectrum of SCy7 and the maximum extinction coefficient, as stated by the manufacturer (240,600 L·mol^–1^ cm^–1^), and was then fixed to 2.48 × 10^–16^ cm². In this fitting, we kept *k*_iso_, *k*_th1_, and *k*_th2_ global and fixed their values, as obtained from the fitting of 638 nm excitation curves since these parameters should not depend on the excitation wavelength, while σ_biso1_ and σ_iso2_ were fitted with a scaling factor, *F*, to the fitted cross-sections at 638 nm excitation since they should both scale with  and the difference in  for different excitation wavelengths. σ_biso2_ was fitted globally. *Q* was fitted globally for all irradiances but individually between the two emission ranges. Also this fit resulted in curves well in agreement with the experimental TRAST curves, with the following globally fitted cross-section values: σ_biso1_(785 nm) = *F* × 0.012 × 10^–16^ cm^2^, σ_iso2_(785 nm) = *F* × 0.2 × 10^–16^ cm^2^ and σ_biso2_(785 nm) = 2.9 × 10^–16^ cm^2^, with *F* fitted to 14 and thus a correspondingly larger excitation cross-section  (and of σ_biso1_ and σ_iso2_) at 785 nm versus at 638 nm excitation. σ_biso2_ was fitted to be 14.5 times higher at 785 nm than at 638 nm excitation, indicating that the excitation spectrum of *P*_2_ is redshifted, similar to that of *P*_1_. *Q* was fitted to 4.6 for the R-filter and to 5.6 for the FR-filter. The higher fitted values of σ_biso1_, σ_iso2_, and σ_biso2_ at 785 nm excitation compared to at 638 nm excitation are consistent with and reflect the higher excitation cross sections of *P*_1_ and *P*_2_ at 785 nm. This is also a likely reason for the higher *Q* value at 785 nm excitation, together with a redshifted emission of *P*_2_ compared to *N*. Given that all SCy7 fluorophores are in the *N* state at the onset of excitation (as assumed, eq S5), a *Q* > 1, as obtained, is a prerequisite for upward, inverse relaxation observed in the experimental TRAST curves (Figure 1C, red and purple data points).

Taken together, fitting of a limited number of parameter values to a three-state photoisomerization model for SCy7 with two emissive states (Figure 3A) could well incorporate all major observations in the FCS and TRAST measurements and was consistent over all different excitation and emission wavelength regions. Figure 3B,C shows the simulations of how the populations of the *N*, *P*_1_, and *P*_2_ states evolve upon 638 and 785 nm excitation, respectively. The simulations generated based on the fitted parameter values and considering different excitation intensities applied further illustrate how the underlying state population kinetics contribute to the observed relaxations in the TRAST experiments. Figure 3D shows how the *N*, *P*_1_, and *P*_2_ state populations depend on Φ_exc_ at CW excitation. It can be noted that for Φ_exc_< 50 kW/cm^2^, the populations are clearly Φ_exc_-dependent, while for higher Φ_exc_, no major effects on the steady state are found. At Φ_exc_ approaching 1000 kW/cm^2^ however, excited-state saturation effects set in, particularly in *N* (having the longest excited-state lifetime).

It can be noted that the prominent build-up of photoisomerized states, as observed for SCy7 (Figure 1B,C), does not necessarily require a significant Φ_iso_. This build-up is determined by the balance between the effective isomerization and back-isomerization rates (Figure 3A, eqs S2–S4). If these rates (and corresponding isomerization and back-isomerization quantum yields) are low in both directions, prominent populations of photoisomerized states are possible under continuous excitation, even in heptacyanine dyes with smaller Φ_iso_. For reference, we performed FCS measurements of the heptamethine dye hexamethylindotricarbocyanine iodide (HITCI), lacking the ring-substitution of SCy7 in between the head groups (Figure S4A). HITCI has been reported to have an insignificant Φ_iso_, with excited-state deactivation dominated by solvent-mediated decay rates.^27^ In FCS curves recorded from HITCI in aqueous solution under 638 nm excitation (Figure S4B), we observe a similar, prominent relaxation term as for SCy7 under similar conditions (Figure 1B). As for SCy7 (Figure S1A), the amplitude of this relaxation term is also higher in FCS curves from HITCI (Figure S4B), with the B-filter used instead of the R-filter. Moreover, in FCS curves recorded from HITCI with NIR excitation (760 nm) and using a corresponding NIR emission filter, only a very small isomerization amplitude was observed.^27^ This is also what we observe for SCy7 under corresponding NIR excitation conditions (Figure S1B). Taken together, this indicates that formation of a redshifted emissive photoisomerized state can take place not only in SCy7 but seems to be a more general feature in heptamethine cyanine dyes and that prominent populations of photoisomerized states (emissive or not) can be generated in these dyes under steady-state excitation conditions, also if they have a low Φ_iso_.

### Environmental Effects on the Photoisomerization

Next, we investigated the validity of the model of Figure 3A and how the photoisomerization properties of SCy7 were influenced by different environmental conditions. First, TRAST curves recorded from SCy7 in different alcohols under otherwise identical experimental conditions (785 nm excitation, same Φ_exc_ and R-filter) revealed prominent effects in the overall TRAST relaxations (Figure 4A). While the overall amplitudes were negative for PBS, as well as for methanol, they turned positive and steadily increased for higher alcohols, an effect likely coupled to the polarity of the solvents (inset Figure 4A). The relaxation times of the TRAST curves likewise followed a clear trend, increasing with higher viscosities of the solvents (inset Figure 4A). The viscosity dependence agrees well with previous photoisomerization studies of cyanine fluorophores in general^35,44^ and was similarly observed also for SCy7 in PBS with different concentrations of sucrose added to change the solvent viscosity (Figure S5). In the absorption spectra recorded from SCy7 in the different alcohol solvents (Figure S6) as well as in the emission spectra (data not shown), we observed a shift toward longer wavelengths for higher alcohols (lower solvent polarities). The shifts in these spectra, attributed to the all-*trans* state, *N*, can likely also be accompanied by shifts of the spectra of *P*_1_ and *P*_2_. With the excitation wavelength and emission filter used in the TRAST experiments, the spectral shifts found with higher alcohols (Figure S6) can favor the brightness of *N* over that of *P*_2_, resulting in lower *Q* values for these solvents and can thereby lead to the observed effects on the relaxation amplitudes in the TRAST curves shown in Figure 4A. Further support for this interpretation was found from fluorescence decay measurements by TCSPC (Figure S7A–D). At 638 nm excitation and using the B-filter, the fluorescence decay of SCy7 in the different alcohols could be fitted to a monoexponential decay (Figure S7A), while a biexponential decay model was required at 785 and 638 nm excitations using the R-filter (Figure S7B,C). In these experiments and as argued above for SCy7 in PBS, the first (longer) lifetime can be attributed to the *N* state and the second (shorter) one to the *P*_2_ state. The relative amplitude of the second lifetime component, , was found to decrease with higher alcohols (Table S1), which indicates a lower *Q* and that *P*_2_ is formed to a lesser extent in these solvents, consistent with the TRAST data in Figure 4A. We also found (from SCy7 in methanol, 638 nm excitation, R-filter, Figure S7D) that  increased with higher Φ_exc_ applied, suggesting an increased *P*_2_ population, in agreement with the experimental observations from TRAST (Figure 1C) and spectral-TRAST (Figure 2D) measurements of SCy7 in PBS. In the lifetime measurements, both the short and long lifetimes were found to increase with higher alcohols (Table S1). This increase can partly be explained by their higher viscosities, which slow down the isomerization (as a channel of excited state decay), thereby also favoring emissive excited-state relaxations. However, nonradiative decay rates of excited-state heptacyanine dyes have been found to mainly depend on other solvent-mediated effects, particularly on polarity and hydrogen-bond assisted contributions.^27^ Since the effective isomerization and back-isomerization rates (*k*_iso1_´, *k*_iso2_´, *k*_biso1_´, and *k*_biso2_´, as given in eqs S3A–C and S4A,B) are mainly excitation-driven, the populations of the *N*, *P*_1_, and *P*_2_ states and their kinetics also largely depend on the excitation and de-excitation rates of these states. We can therefore expect significant differences in the TRAST curves of Figure 4A,C to be due to a combination of solvent viscosity and polarity effects, as well as hydrogen-bond assisted contributions to the different excited-state decays of the dyes.

To further investigate the influence of hydrogen bond-assisted deactivation of the excited states, we also performed TRAST, TCSPC, and spectrofluorometer measurements of SCy7 in DMSO. DMSO has a similar polarity to water but is highly aprotic, that is, more polar but less protic than any of the alcohols.^50^ In DMSO, SCy7 displayed a longer fluorescence lifetime (Figure S7A) and a more redshifted absorption spectrum (Figure S6) compared to water and any of the alcohols. Moreover, while TRAST curves recorded from SCy7 in DMSO displayed isomerization relaxation times close to those found in propanol and butanol (with similar viscosities as DMSO), the recorded isomerization amplitudes were larger than in any of the alcohols (Figure 4A). This indicates that the aprotic character of a solvent, described by its logarithmic autoprotolysis constant *pK*_AP_, is together with its polarity (dielectric constant) and viscosity also a major parameter influencing the isomerization kinetics under steady-state excitation. This then also influences the mainly excitation-driven population balance between the different isomerization states.

Next, we investigated the applicability of the three-state photoisomerization model (Figure 3A) to alcohol solvents by a series of TRAST curves recorded from SCy7 in methanol and ethanol at different Φ_exc_ and using the two different excitation wavelengths (638 and 785 nm) and emission filters (B- or R-filters). The experimental curves from the methanol and ethanol solvents were globally fitted to the same model following the same procedure as for the TRAST curves recorded in PBS solution (Figure 1C). This global fitting procedure could well reproduce the experimental data (Figure S8A,B), with fitted parameter values (Table S3) comparable to the ones found for SCy7 in aqueous solution. Notably, however, the fitted *Q* values were generally lower than in water, which is well in line with the observations from the spectral (Figure S6) and TCSPC measurements (Figure S7).

As a further investigation of how fluorophore-solvent interactions affect the photoisomerization properties, we also studied amino-Cy7, a modified version of SCy7 (Figure 4B). TRAST curves recorded from amino-Cy7 in PBS, in different alcohols, and in DMSO (Figure 4C) show the same trend as those from SCy7 (Figure 4A), that is, decreased negative relaxation amplitudes in more aprotic, higher alcohols with lower solvent polarities and a clear decay amplitude for amino-Cy7 in DMSO (inset, Figure 4C). A similar trend was also found in the absorption spectra of amino-Cy7, which were increasingly redshifted with lower solvent polarities (Figure S9). Notably, however, for amino-Cy7, the TRAST relaxation amplitudes were negative for all solvents, indicating generally higher *Q* values, that is, a relatively higher brightness of *P*_2_ compared to *N* in amino-Cy7, compared to SCy7 under the same experimental conditions. This difference between amino-Cy7 and SCy7 shows that minor differences in the molecular polarity of a cyanine fluorophore and in its sidechain can lead to relatively large differences in their photoisomerization properties, as observed via TRAST measurements. Vice versa, the photoisomerization properties of these fluorophores are also quite sensitive to the immediate polarity, viscosity, and solvent conditions around the fluorophores.

**Figure 5:**
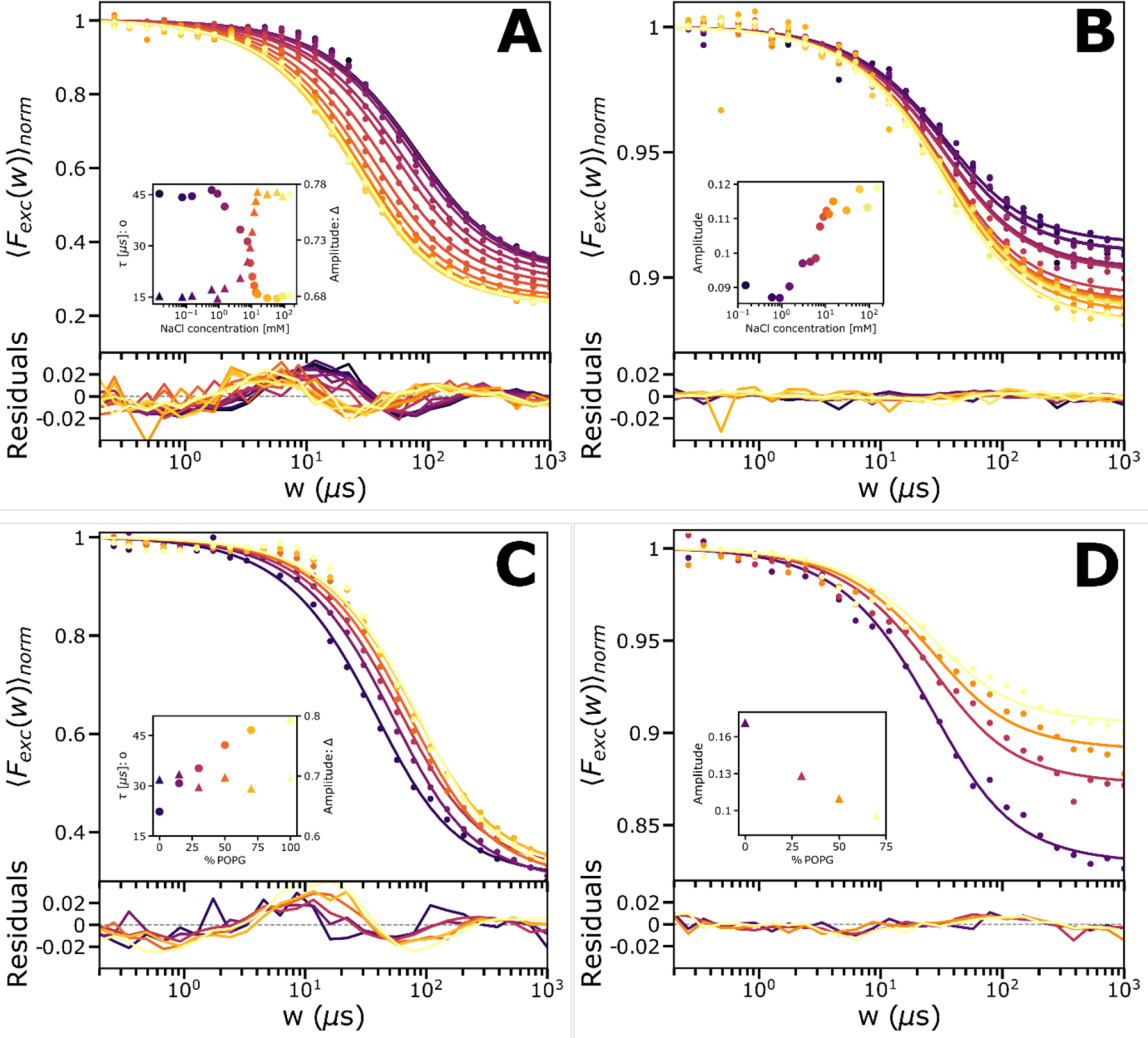
(A–D) TRAST curves from SCy7-labelled POPE-lipids included in SUVs in PBS solution (12 mM, pH 7.2). The SUVs were made from POPC (zwitterionic head group) and POPG (negatively charged head group) in different proportions and with different salt (NaCl) concentrations added. Experimental data are represented by dots. The TRAST curves were individually fitted to a one-exponential relaxation model (solid lines) for the population change in SCy7 upon onset of excitation, with the amplitude and relaxation time as the only fitted parameters. Fitting residuals plotted below. (A) TRAST curves recorded from SCy7 in SUVs with 70% POPG and 30% POPC and with varying NaCl concentrations. Excitation at 638 nm (4.1 kW/cm^2^), emission detected with the R-filter. Both the amplitude and the relaxation time were fitted as free parameters. Inset: fitted amplitude and relaxation time versus salt concentration. For SUVs made of 100% POPC, no sensitivity to salt concentration was observed (data not shown). (B) TRAST curves recorded from the same samples as in (A) but at 785 nm excitation (3.7 kW/cm^2^) excitation and with emission detected with the R-filter. The relaxation time for all curves was globally fitted to 20 μs, while the amplitudes were freely fitted. Inset: fitted amplitudes versus salt concentration, showing a sensitivity in the same range as in (A); 1–20 mM. (C) TRAST curves recorded from SUVs made of POPC and POPG in different proportions. Excitation at 638 nm (4.9 kW/cm^2^), emission detected with the B-filter. Both the amplitude and the relaxation time were fitted as free parameters. Inset: fitted amplitudes and relaxation times versus fraction of POPG in the SUVs. (D) TRAST curves recorded from the same samples as in (C), but at 785 nm excitation (4.6 kW/cm^2^) excitation and with emission detected with the R-filter. Here, the relaxation time was again fitted globally, while the relaxation time was fitted freely. Inset: fitted amplitudes versus fraction of POPG in the SUVs.

To further illustrate how the photoisomerization properties of SCy7 are affected by the immediate environment, we recorded TRAST curves from SUVs with SCy7-labelled POPE, with the SUVs made from POPC (zwitterionic head group) and POPG (negatively charged head group) in different proportions and with different salt (NaCl) concentrations added into the SUV solutions (Figure 5A–D). At 638 nm excitation and using the B-filter (Figure 5A,C), we find that the overall photoisomerization relaxation time increases with higher polarity at the membrane surface (with lower concentrations of NaCl shielding the charges, or higher fractions of POPG versus POPC). With 638 nm excitation and the B-filter, *Q* = 0 and we can expect the TRAST curves to reflect transitions to and from *N*. The slower transitions observed with higher local polarity are likely a consequence of larger electrostatic interactions between SCy7 and the lipid membrane. This is supported by the observation that for SUVs made of 100% POPC, no sensitivity to salt concentration was observed (data not shown). At 785 nm excitation and using the R-filter, fluorescence from both *N* and *P*_2_ contributes to the recorded TRAST curves (Figure 5B,D), in which case the decay in *N* upon onset of excitation is balanced with an increase in *P*_2_, resulting in lower amplitudes in these curves. In contrast to the TRAST curves recorded at 638 nm excitation (Figure 5A,C), altered local polarity by changed POPG contents or NaCl concentrations had no significant effect on the relaxation time, which could be globally fitted for each set of TRAST curves in Figure 5B,D, respectively. This is likely due to a faster exchange between the *P*_1_ and *P*_2_ states than between *N* and *P*_1_, with the faster *P*_1_–*P*_2_ exchange then largely reflected in the relaxation time of the TRAST curves. The lower relaxation amplitudes with higher polarity follow the same trend as observed in the alcohol measurements (Figure 4A,C) and may thus likewise be attributed to higher *Q* values with increased polar environments.

## Concluding Remarks

By a combination of TRAST, FCS, spectrofluorometric, and fluorescence lifetime measurements, we have identified a photoisomerized, redshifted emissive state in SCy7. The population dynamics, as observed applying different Φ_exc_ and using different excitation wavelengths and emission spectral ranges, can be described with a three-state photoisomerization model, including an emissive all-*trans* (*N*), a dark photoisomerized mono-*cis* (*P*_1_) and a redshifted emissive photoisomerized state (*P*_2_). This model is consistent with several studies on cyanine dyes,^15,17,28,51,52^ in which the photoisomerized mono-*cis* conformation has been found to show very low fluorescence, which suggests that the additional red-emissive state we observe then should represent an additional photoisomerized state. Moreover, for SCy7 and other heptamethine cyanine dyes, there are several positions along the conjugated hydrocarbon chain where isomerization can take place, which further supports this model, with an additional photoisomerized state being formed. However, the fitted parameter values for this model (Table 1) infer that the transitions between *P*_1_ and *P*_2_ are much faster than those between *N* and *P*_1_. On the time scale of the *N*–*P*_1_ transitions, *P*_1_ and *P*_2_ can thus be observed as a single, time-averaged state, with a lower brightness than *P*_2_. Consequently, our experimental TRAST and FCS data can also be fitted to a simpler two-state isomerization model, including an all-*trans* (*N*) and mono-*cis* state (*P*_1_), in which *P*_1_ is no longer dark but has a redshifted emission (Figure S10). Moreover, in the parameter fitting to the experimental FCS and TRAST curves, there is a strong cross-covariance between the brightness parameter *Q* and the population of *P*_2_ (or *P*_1_), making several combinations of these parameters almost as consistent with the experimental data. Although additional studies will be needed to fully resolve the underlying photoisomerized states of *P*_1_ and *P*_2_, the implications following the occurrence of an additional redshifted emissive state in SCy7 are in several aspects still the same, irrespective of the actual states behind. Reference measurements on HITCI (Figure S3B) further suggest that significant build-up of photoisomerized states under moderate steady-state excitation is not specific for SCy7 nor is the formation of redshifted emissive photoisomers. This rather seems to be properties SCy7 has in common with other heptamethine cyanine dyes. Generally, the more detailed view of the photoisomerization kinetics of SCy7 and NIR cyanine dyes, as provided in this work, and how the populations of *P*_1_ and *P*_2_ depend on Φ_exc_, excitation and emission wavelengths and sample conditions, such as polarity, viscosity and steric constraints, will help the design of SMD and SRM experiments in the NIR to avoid effects of such kinetics. For SMLM with cyanine dyes, it has recently been reported that photoisomerized states of Cy5 with redshifted excitation spectra may lead to unwanted FRET-mediated excitation transfer between closely (<10 nm) located non-photoisomerized and photoisomerized fluorophores.^36^ Thus, in the context of SMLM, the fact that such photoisomerized states also can show redshifted emission, as very recently shown for Cy5,^53^ and shown for NIR cyanines in this work, makes it necessary to also account for this emission source in the analyses of the resulting blinking. Beyond SMD and SRM experiments, since the redshifted emissive state is generated already at relatively low excitation intensities (Figure 3D), excitation and emission spectra of NIR cyanine dyes can be expected to be altered in a range of experiments. This can be useful to account for in experiments relying on the shape of these spectra, such as in FRET and in multicolor experiments using linear spectral unmixing. On the other side, fluorophores emitting further into the NIR are scarce.^54,55^ In this context, knowledge about the photodynamics of this additional red-emissive state and on how it can be promoted may be used as a strategy to push the emission of NIR cyanines further into the NIR. Similarly, promotion of this state may also provide an approach to enhance photosensitization of nanoparticles with absorption spectra further into the NIR. The NIR emission generated by FRET by closely located cyanine fluorophores can also reflect inter- and intramolecular distances. Finally, the strong effects from the local viscosity, polarity, and solvent molecule interactions on the photoisomerization kinetics of SCy7 and on the formation of its redshifted photoisomer also suggest the use of SCy7 and other NIR cyanine dyes as environmental sensors. Such environmental information can be monitored by TRAST in the NIR with low autofluorescence and scattering conditions and on a broad range of samples and experimental conditions.

**Table 1:** Fitted Parameter Values for SCy7 in PBS to Data Measured Either by FCS at 638 nm Excitation or by TRAST at Either 638 nm or 785 nm Excitation.^a^

	FCS(638 nm)	TRAST (638 nm)	TRAST (785 nm)	unit
*k*_iso1_	12.5	14	14	μs^–1^
σ_biso1_	0.011 × 10^–16^	0.012 × 10^–16^	14 × 0.012 × 10^–16^	cm^2^
σ_iso2_	0.3 × 10^–16^	0.2 × 10^–16^	14 × 0.2 × 10^–16^	cm^2^
σ_biso2_	0.13 × 10^–16^	0.2 × 10^–16^	2.9 × 10^–16^	cm^2^
*k*_biso1_^Th^		0.034	0.034	μs^–1^
*k*_biso2_^Th^		0.011	0.011	μs^–1^
*Q*		0.56 (R-filter)	4.6 (B-filter) 5.6 (R-filter)	

a In the table,  is the relative detected brightness of *P*_2_ compared to *N*, where ^1^*q*_*D*_ and ^2^*q*_*D*_ denote the overall detection quantum yields of the emission from the excited singlet state of *N* and *P*_2_, respectively, and ^1^*q*_*F*_ and ^2^*q*_*F*_ are the fluorescence quantum yields of these states. σ_*N*_ and  denote the excitation cross section of the ground singlet state of *N* and *P*_2_, respectively; see also eq 1.

## Data Availability

All relevant raw data behind this study are available via DOI: 10.5281/zenodo.7732900.

## Supporting Information Available

Section 1: Electronic state model for SCy7. Section 2: Spatial distribution of excitation rates, calculation of average rates. Figure S1: FCS curves recorded from SCy7 in PBS solution. Figure S2: TRAST curves recorded from SCy7 in PBS solution. Figure S3: TCSPC fluorescence decay measurements of SCy7 in PBS solution. Figure S4: Structure of HITCI and FCS curves recorded from HITCI. Figure S5: FCS curves from SCy7 in PBS with sucrose. Figure S6: absorption spectra of SCy7 in different solvents. Figure S7: TCSPC fluorescence lifetime measurements of SCy7 in different solvents. Figure S8: Experimental and fitted TRAST curves from SCy7 in methanol and ethanol. Figure S9: Absorption spectra of amino-Cy7 in different solvents. Figure S10: TRAST and FCS data from SCy7 in PBS fitted to a two-state isomerization model. Table S1: Fitted parameter values from TCSPC fluorescence decay measurements of SCy7 in different solvents. Table S2: Fitted A_P2_ amplitudes from the fluorescence decay data. Table S3: Fitted parameters from FCS and TRAST curves in Figure S10 (PDF)
