# Supplementary material for: Photoisomerization of Heptamethine Cyanine Dyes Results in Red-Emissive Species: Implications for Near-IR, Single-Molecule, and Super-Resolution Fluorescence Spectroscopy and Imaging
Source: J Phys Chem B. 2023 Apr 3;127(14):3208–22. doi: 10.1021/acs.jpcb.2c08016 (PMC10108366; doi:10.1021/acs.jpcb.2c08016)
Supplement: Supplementary file 1 — jp2c08016_si_001.pdf [file jp2c08016_si_001.pdf]

Supplementary part

Photo-Isomerization of Heptamethine Cyanine Dyes Results in Red Emissive Species – Implications for Near-IR, Single-Molecule and Super-Resolution Fluorescence Spectroscopy and Imaging

Elin Sandberg[a], Joachim Piguet[a], Uliana Kostiv[a], Glib Baryshnikov[b], Haichun Liu[a], Jerker Widengren[a,*]

[a] Royal Institute of Technology (KTH), Experimental Biomolecular Physics, Dept. Applied Physics, Albanova Univ Center 106 91 Stockholm, Sweden

[b] Linköping University, Dept. Science and Technology, Campus Norrköping, 601 74 Norrköping, Sweden

* Corresponding author: Email: jwideng@kth.se, Phone: +46-8-7907813

Section S1. Electronic state model for SCy7

With the electronic state model shown in Figure 4A, the electronic state population dynamics of a SCy7 fluorophore, subject to a constant excitation photon flux of Φ_{exc} starting at time t=0, is given by

$$\frac{d}{dt}\bar{A}(t) = M \cdot \bar{A}(t) \qquad \text{(S1)}$$

Here, $\bar{A}(t) = [\ [N](t), [P_1](t),\ [P_2](t)]^T$ represents the population probabilities of the all-*trans*, a mono-*cis*, and a double-*cis* state and

$$M = \begin{bmatrix} -k_{iso1}{}' & k_{biso1}{}' & 0 \\ k_{iso1}{}' & -k_{biso1}{}' - k_{iso2}{}' & k_{biso2}{}' \\ 0 & k_{iso2}{}' & -k_{biso2}{}' \end{bmatrix} \qquad \text{(S2)}$$

is the model matrix describing the transitions between the states. In the matrix, the effective isomerization rates, from N to P_1, and from P_1 to P_2, are given by:

$$k_{iso1}{}' = k_{iso1} \cdot \frac{\sigma_N \cdot \Phi_{exc}}{\sigma_N \cdot \Phi_{exc} + k_{10}^N} \qquad \text{(S3A)}$$

$$k_{iso2}{}' = k_{iso2} \cdot \frac{\sigma_{P_1} \cdot \Phi_{exc}}{\sigma_{P_1} \cdot \Phi_{exc} + k_{10}^{P_1}} = \left\{k_{10}^{P_1} \gg \sigma_{P_1} \cdot \Phi_{exc}\right\} = \sigma_{iso2} \cdot \Phi_{exc} \qquad \text{(S3B)}$$

with σ_N and σ_{P_1} denoting the excitation cross sections of the singlet ground state of N and P_1, respectively, and with k_{10}^N and $k_{10}^{P_1}$ signifying the decay rates from the excited singlet state to the ground singlet state, in N and P_1 respectively. Since k_{iso2}, $k_{10}^{P_1}$ and σ_{P_1} could not be individually determined, we defined the isomerization from P_1 to P_2 as an isomerization cross section:

$$\sigma_{iso2} = k_{iso2} \cdot \frac{\sigma_{P_1}}{k_{10}^{P_1}} \qquad \text{(S3C)}$$

Similarly, the effective back-isomerization rates from P_1 to N, and from P_1 to P_2, are given by: $k_{biso1}{}' = k_{biso1} \cdot \frac{\sigma_{P_1} \cdot \Phi_{exc}}{\sigma_{P_1} \cdot \Phi_{exc} + k_{10}^{P_1}} + k_{biso1}^{Th} = \left\{k_{10}^{P_1} \gg \sigma_{P_1} \cdot \Phi_{exc}\right\} = \sigma_{biso1} \cdot \Phi_{exc} + k_{biso1}^{Th}$ (S4A)

$$k_{biso2}{}' = k_{biso2} \cdot \frac{\sigma_{P_2} \cdot \Phi_{exc}}{\sigma_{P_2} \cdot \Phi_{exc} + k_{10}^{P_2}} + k_{biso2}^{Th} = \left\{k_{10}^{P_2} \gg \sigma_{P_2} \cdot \Phi_{exc}\right\} = \sigma_{biso2} \cdot \Phi_{exc} + k_{biso2}^{Th} \qquad \text{(S4B)}$$

With corresponding back-isomerization cross sections defined as:

$$\sigma_{biso1} = k_{biso1} \cdot \frac{\sigma_{P_1}}{k_{10}^{P_1}} \qquad \text{(S4C)}$$

$$\sigma_{biso2} = k_{biso2} \cdot \frac{\sigma_{P_2}}{k_{10}^{P_2}} \qquad \text{(S4D)}$$

The initial condition for Eq. (S1) is

$$\overline{A}(0) = [1\ 0\ 0\ ]^T \qquad \text{(S5)}$$

, assuming all SCy7 fluorophores are in the singlet (ground) state before onset of excitation at $t = 0$.

For a rectangular excitation pulse, Φ_{exc} is constant throughout the excitation duration and the matrix M is not time dependent. The general solution to Eq S1 is then

$$\overline{A}(t) = e^{Mt} \cdot \overline{A}(0) \qquad \text{(S6)}$$

The dependence of the detected fluorescence at time, t, after onset of excitation is then given by

$$F(t) = {}^1q_F \cdot {}^1q_D \cdot k_{10}^N \cdot \frac{\sigma_N \cdot \Phi_{exc}}{\sigma_N \cdot \Phi_{exc} + k_{10}^N} \cdot [N](t) + {}^2q_F \cdot {}^2q_D \cdot k_{10}^{P_2} \cdot \frac{\sigma_{P_2} \cdot \Phi_{exc}}{\sigma_{P_2} \cdot \Phi_{exc} + k_{10}^{P_2}} \cdot [P_2](t) \qquad \text{(S7)}$$

, with σ_X denoting the excitation cross section, ${}^Xq_F$ the fluorescence quantum yield and ${}^Xq_D$ the detection quantum yield of the emission from N (X=1) and P_2 (X=2) state, respectively. For the excitation conditions in our study, $k_{10} \gg \sigma_N \cdot \Phi_{exc}, \sigma_{P_2} \cdot \Phi_{exc}$, so that we can assume

$$F(t) = {}^1q_F \cdot {}^1q_D \cdot \sigma_N \cdot \Phi_{exc} \cdot \left([N](t) + Q \cdot [P_2](t)\right) \qquad \text{(S8)}$$

, with $Q = ({}^2q_F \cdot {}^2q_D \cdot \sigma_{\mathrm{N}})/({}^1q_F \cdot {}^1q_D \cdot \sigma_{P_2})$ representing the relative brightness of N, compared to P_2.

Section S2. Spatial distribution of excitation rates, calculation of average rates

The excitation beam does not form a uniform profile, and the excitation photon flux, $\Phi_{exc}(\bar{r})$, is thus a function of position in the sample. As a consequence, our TRAST analysis included a spatial dependence on both the excitation rates and the resulting electronic state populations. The total fluorescence signal on each pixel of the camera then becomes a convolution of $[N](t) + Q \cdot [P_2](t)$ and the microscope collection efficiency function, $CEF(\bar{r})$. While simulating the whole 3D sample volume, and computing the projected 2D image on the camera, we found that pre-computing an average observed excitation rate, $\hat{k}_{01}$, for each ROI to be analyzed, speeds up the fitting significantly, without appreciable loss of accuracy. The approximate $\hat{k}_{01}$ is computed once, before fitting starts, by weighting $k_{01}(\bar{r})$ by brightness and collection efficiency, $CEF(\bar{r})$, in the following manner

$$\hat{k}_{01} = \frac{\iiint k_{01}(\bar{r}) \cdot \hat{S}_1(\bar{r}) \cdot CEF(\bar{r}) dV}{\iiint \hat{S}_1(\bar{r}) \cdot CEF(\bar{r}) dV} \qquad (S9)$$

$\hat{S}_1(\bar{r}) = k_{01}(\bar{r})/(k_{10} + k_{01}(\bar{r}))$ represents the population of excited singlet state SCy7 when in an all-*trans* form, N, at onset of excitation, after equilibration between the ground and excited singlet states of N, but before build-up of the other states.

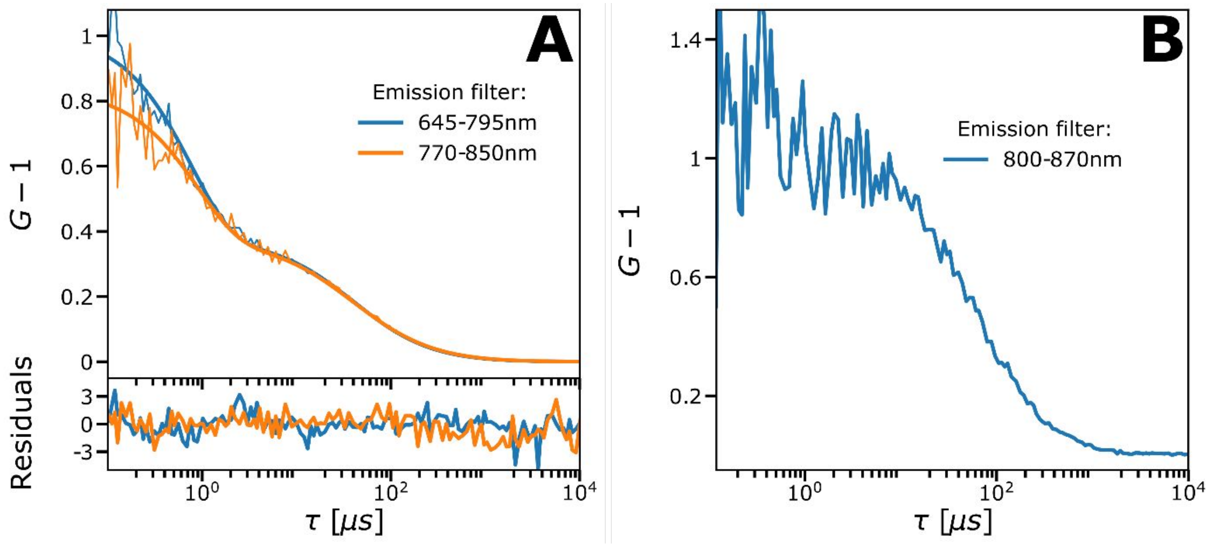

Figure S1. *FCS curves recorded from SCy7 in PBS solution (12mM, pH 7.2)*

(A): FCS curves recorded at 638nm excitation with Φ_{exc} *280*kW/cm², with the fluorescence detected in *different emission ranges, as indicated in legend. Fitted curves to a two-state model (Eq. 7) are shown as thick solid lines with fitting residuals below. Fitted dark state amplitudes were 0.56 for the red-shifted and 0.63 for the blue-shifted filter.*

(B): FCS curve generated from fluorescence emission in the 800-870nm range, upon 780nm excitation (Φ_{exc} *103kW/cm²). In contrast to excitation at 638nm, almost no dark-state relaxation is observed.*

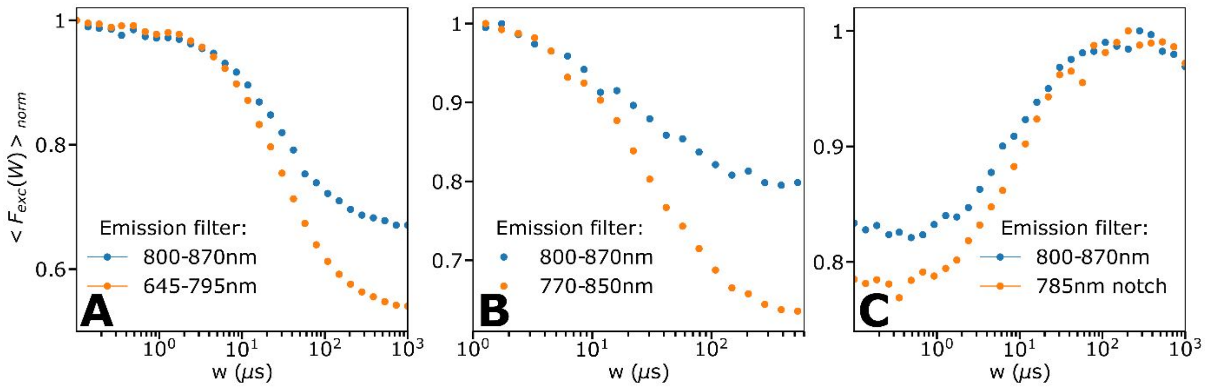

Figure S2. *TRAST-curves recorded from SCy7 in PBS solution (12mM, pH 7.2) using different excitation wavelengths and emission filters (transmission specified in legends). Overall, fluorescence detection over longer emission wavelengths resulted in lower relaxation amplitudes in the TRAST curves recorded with 638nm and 730nm excitation, while a larger and inverse amplitude were observed at 785nm excitation, with a larger contribution of longer wavelength fluorescence emission.*

(A): 638nm excitation, Φ_{exc} *3.9kW/cm².*

(B): 730nm excitation, Φ_{exc} *5kW/cm².*

(C): 785nm excitation, Φ_{exc} *4.2kW/cm².*

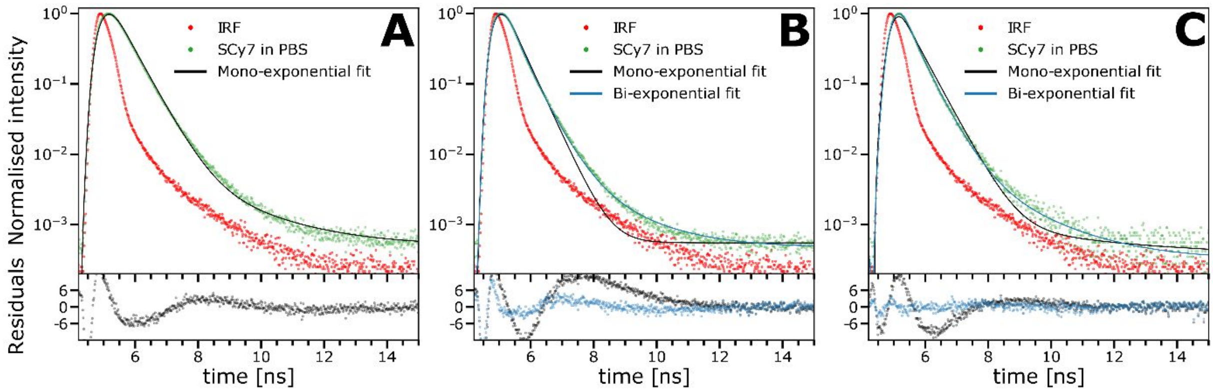

Figure S3. *TCSPC fluorescence decay measurements of SCy7 in PBS solution (12mM, pH 7.2) using different excitation lasers and based on fluorescence detected in different emission ranges. Data is represented by dots. Deconvolution was done based on an instrument response function (IRF) shown in red. Solid lines represent fitted curves, with residuals below.*

(A): Fluorescence decay measured upon 638nm excitation, based on emission collected within 645-795nm. The fluorescence decay could be fitted to a mono-exponential decay, with $\tau_f = 0.52ns$. *Using a bi-exponential decay model for fitting did not improve the residuals significantly (not shown).*

(B): Fluorescence decay measured upon 638nm excitation, based on emission collected within 800-870nm, fitted to a mono-exponential (black line) and a bi-exponential (blue line) decay. In the bi-exponential fit, the lifetime of N was fixed to $\tau_f = 0.52ns$, *as obtained for the blue-shifted emission filter (Figure S3A), and yielded a second fluorescence lifetime component, with* $\tau_{f2} = 0.3ns$ *and a relative amplitude of 53%. This resulted in a better fit to the data than fitting by a single lifetime, mono-exponential decay.*

(C): Fluorescence decay measured upon 780nm excitation, based on emission within 800-870nm, fitted to a mono-exponential (black line) and a bi-exponential (blue line) decay. In the bi-exponential fit, the lifetimes were fixed to $\tau_f = 0.52ns$, $\tau_{f2} = 0.3ns$, *as obtained for 638nm excitation (Figure S3B). This again resulted in a clearly better fit to the experimental data, than by using a single lifetime, mono-exponential decay fitted data poorly. The relative amplitude of the second lifetime component was fitted to 53%.*

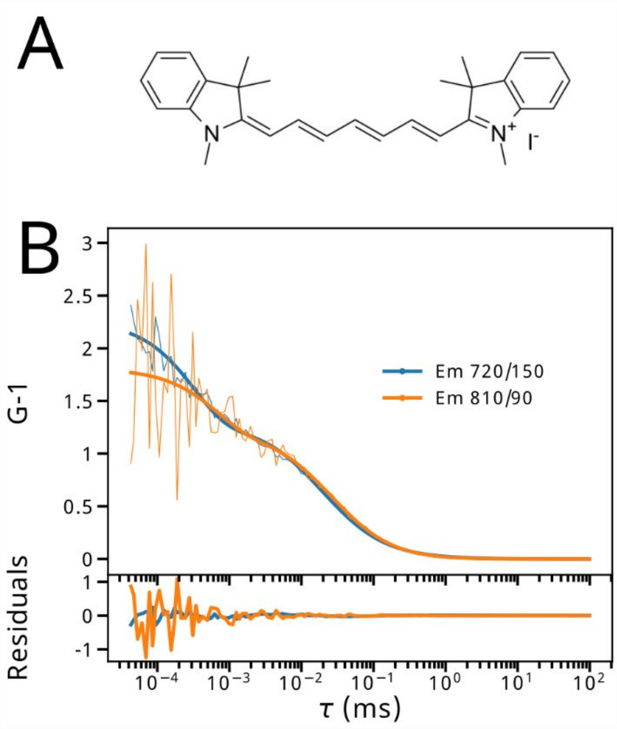

Figure S4.

(A): Structure of the heptamethine dye hexamethylindotricarbocyanine iodide (HITCI).

(B): FCS curves recorded from HITCI in PBS solution (12mM, pH 7.2) at 638nm excitation with Φ_{exc} 200kW/cm², with the fluorescence detected in different emission ranges, as indicated in legend. Fitted curves to a two-state model (Eq. 7) are shown as thick solid lines with fitting residuals below. Fitted relaxation amplitudes / times were 0.30 / 1.1µs for the curve measured with the red-shifted and 0.43 / 0.34µs for curve measured with the blue-shifted filter.

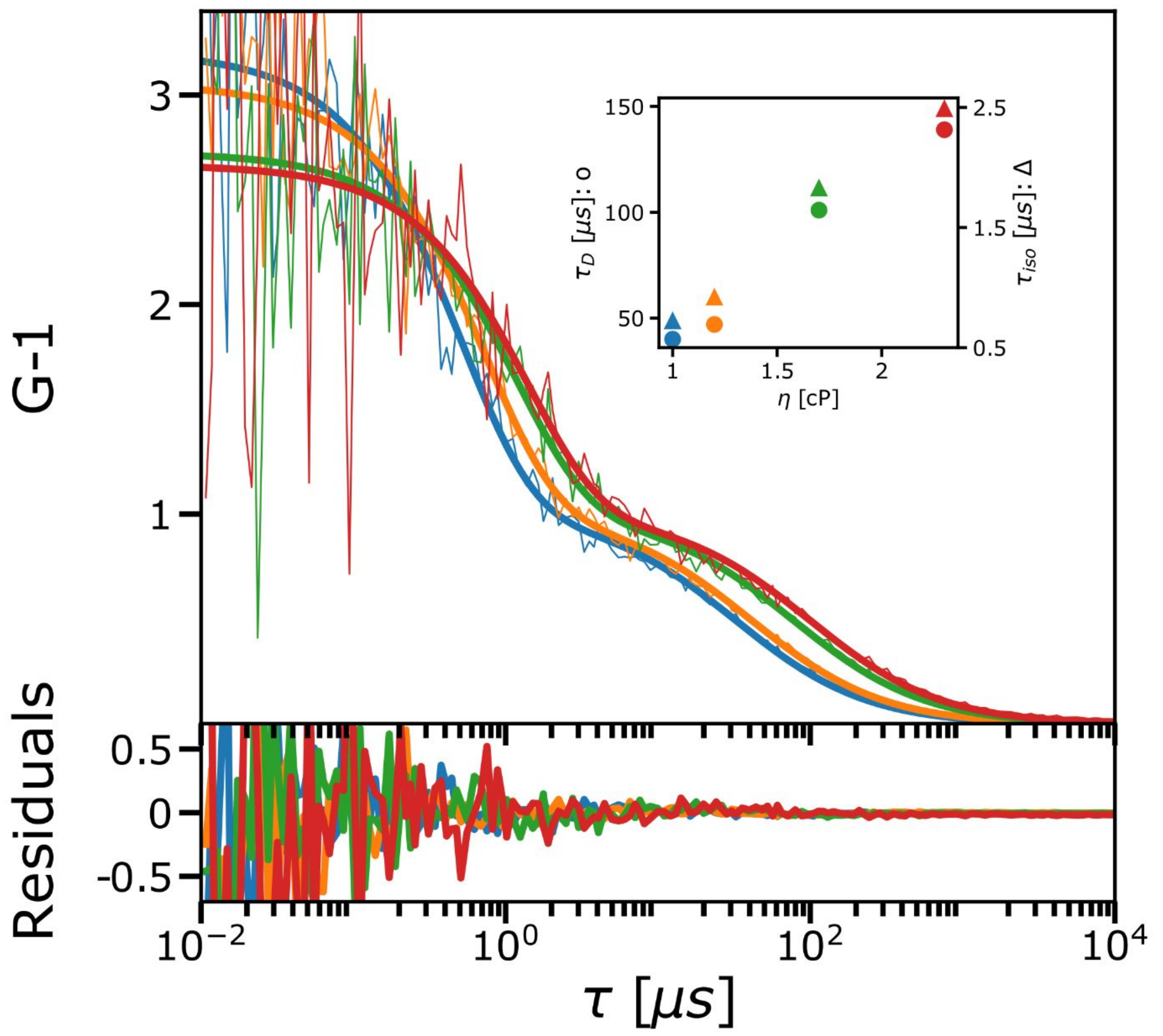

Figure S5. *FCS curves (recorded at 638nm excitation, 470kW/cm², emission detected in the 645-795nm range) from SCy7 in PBS (12mM, pH 7.2) with different concentrations of sucrose added to change the viscosity. The FCS curves were fitted to a two-state isomerization model (Eq. 7). Fitted FCS curves are indicated given by solid thick lines, experimental FCS curves by thinner lines, and fitting residuals below. Inset shows fitted diffusion and isomerization relaxation times versus viscosity, both showing a linear dependence.*

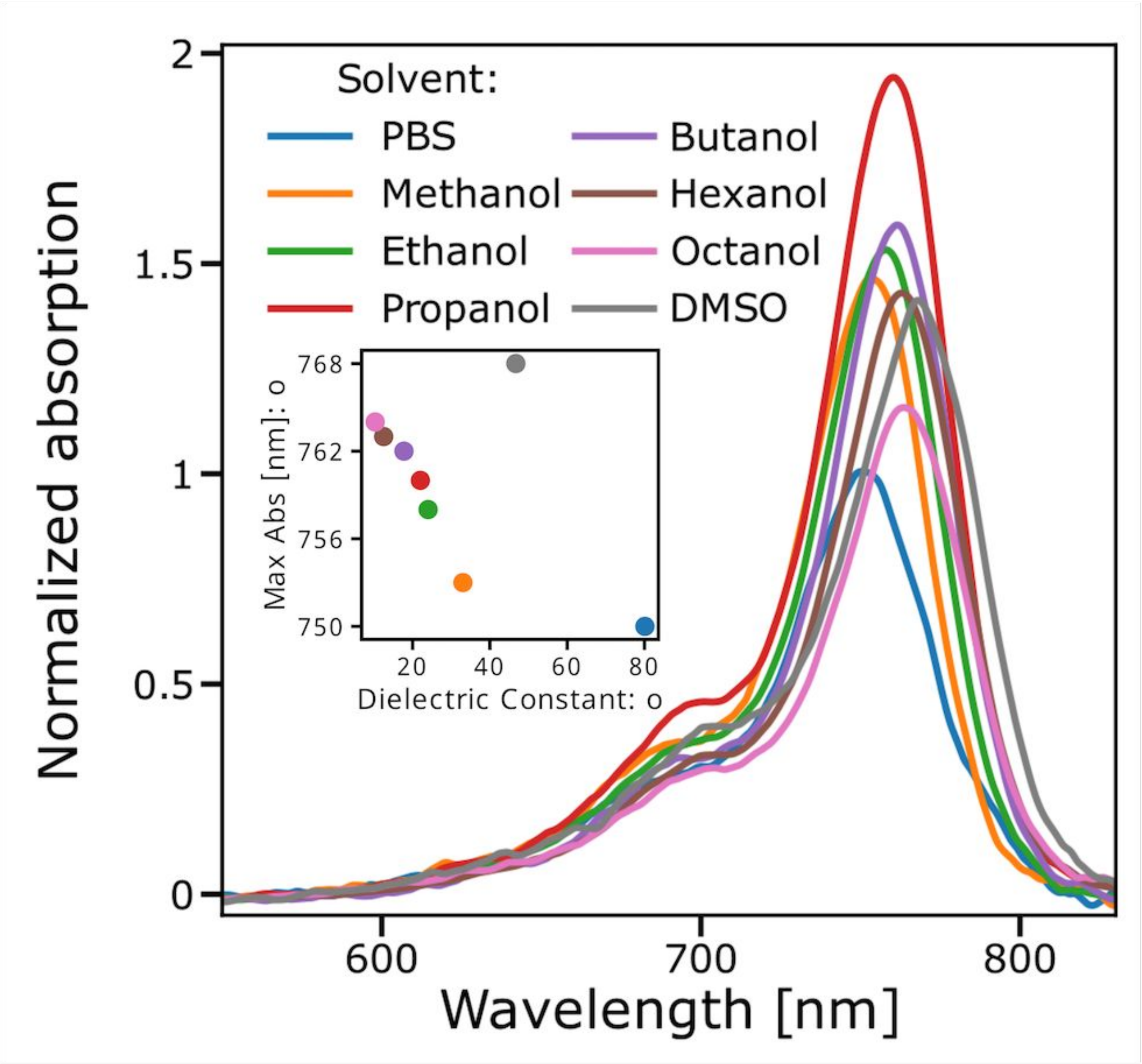

Figure S6. *Absorption spectra of SCy7 in different solvents, normalized with the maximum absorption in PBS. The absorption maximum (*Abs_{max}*) is shifting to longer wavelengths for higher alcohols, likely coupled to their decreased polarities (dielectric constants), as indicated in the inset. The largest red-shift was observed for DMSO however, indicating that the aprotic character of the solvent can also play a major role for the red-shift. Similar to the absorption spectra, the emission maxima were also slightly shifted to longer wavelengths for higher alcohols (data not shown).*

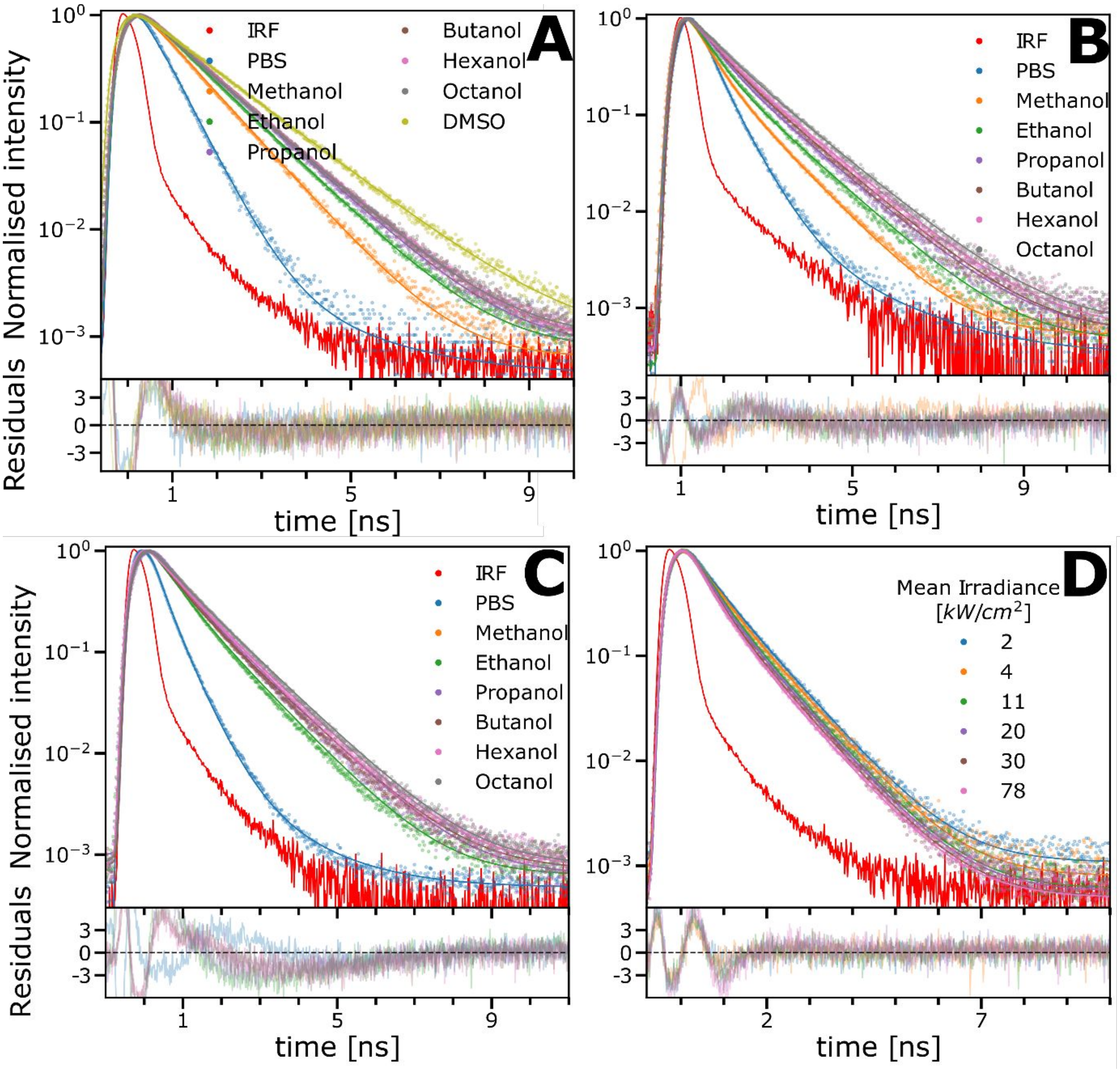

Figure S7. *TCSPC fluorescence lifetime measurements of SCy7 in different solvents. Fluorescence decay data (dots) is deconvolved with the IRF (red line). Solid lines show fitted decay curves, with residuals below.*

(A): Fluorescence decays measured at 638nm excitation (78kW/cm²) and with a 645-795nm emission filter. The data were fitted as a mono-exponential decay, attributed to the decay of the singlet excited state of N, presented as τ_f in Table 1. Generally, τ_f was found to increase with higher alcohols (lower solvent polarities).

(B): Corresponding fluorescence decays measured at 780nm excitation (20 kW/cm²) and with an 800-870nm emission filter. The data were fitted as a bi-exponential decay, with, τ_f fixed according to the fitted values from (A). The second lifetime, τ_{f2}, can be attributed to the decay of the singlet excited state of P_2. Fitted values of τ_{f2} for the different solvents are given in Table S1. Generally, both lifetimes were found to increase with higher alcohols and lower solvent polarities, while the relative amplitude, A_{P2}, of the second exponential (with decay time τ_{f2}) decreased.

(C): Fluorescence decays measured at 638nm excitation (78kW/cm²) with an 800-870nm emission filter. The data were fitted as a bi-exponential decay, with A_{P2} as a free variable, τ_f fixed according to the fitted values from (A) and τ_{f2} fixed to the fitted values from (B). Fitted A_{P2} are listed in Table S1.

(D): Fluorescence decay measurements of SCy7 (1µM) in MeOH at 638nm excitation, with emission detected in the 800-870nm range, and with different excitation intensities applied. The fluorescence decay curves were fitted to a bi-exponential decay, with τ_f and τ_{f2} fixed to the values obtained in (A) and (B) (0.95ns and 0.4ns, respectively), and with A_{P2} fitted freely Fitted A_{P2} values were clearly found to increase with higher excitation intensities applied (Table S2), until an equilibrium between P_2 and N is reached.

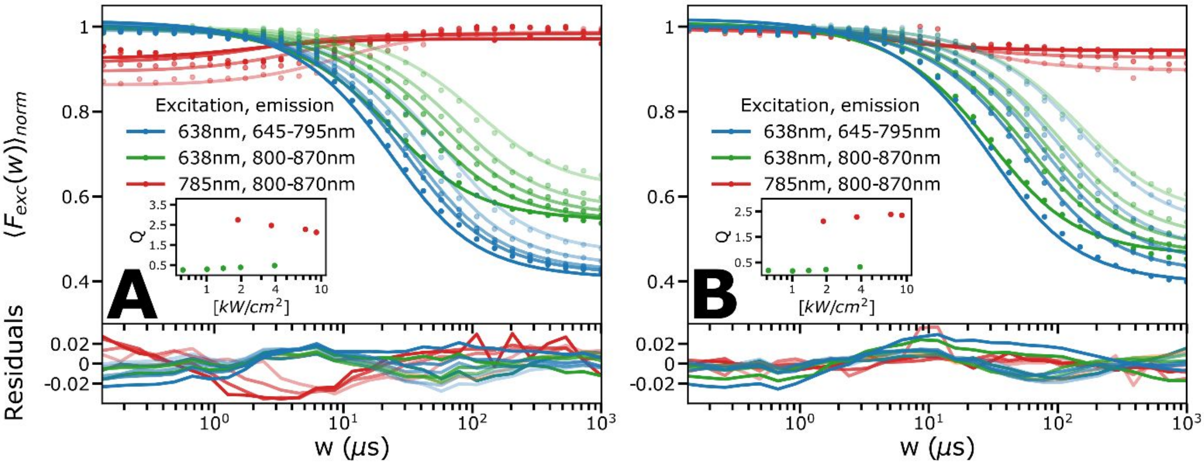

Figure S8. *TRAST curves recorded from SCy7 (1µM) in Methanol (A) and Ethanol (B) using different excitation wavelengths (638nm and 785nm) and emission filters (645-795nm and 800-870nm), as stated in legends. Experimental data is represented by dots and lines show fitted curves (to the double-isomerization model of Figure 3A). The color-intensities display TRAST curves recorded at increasing* Φ_{exc} *[1.9, 3.7, 7.3, 9.1] kW/cm² at 785nm excitation and [0.62, 1.0, 1.4, 2.0, 3.9] kW/cm² at 638nm excitation. Insets show* fitted Q-values *for the different excitation wavelengths and irradiances applied.*

(A): TRAST curves recorded in methanol, fitted to the double-photoisomerization model of Figure 3A, following the same procedure as for the corresponding in PBS (Figure 1B). In the fitting, σ_N *was fixed to 2.14* $\cdot 10^{-16}$*cm² for 638nm excitation and 4.42* $\cdot 10^{-16}$*cm² for 785nm excitation.* k_{10}^{N} *=1/* τ_f*-*k_{iso}*, where* τ_f *was fixed to 0.95ns, as determined by TCSPC (Table S1).* k_{iso1}*,* k_{biso1}^{Th} *and* k_{biso2}^{Th} *were fitted globally for all curves. The fitted curves were found to well reproduce the experimental data, with the following fitted parameter values:* k_{iso}*=8.9µs⁻¹,* k_{th1} *=0.0087µs⁻¹ and* k_{th2}*=0.05µs⁻¹. The cross-sections at 638nm excitation were fitted to* σ_{biso1} = *0.02* $\cdot 10^{-16}$*cm² and* σ_{iso2} = *0.5* $\cdot 10^{-16}$*cm², and were fitted to scale by a factor F=10 to the corresponding cross-sections at 785nm excitation.* σ_{biso2} *was fitted to 0.5* $\cdot 10^{-16}$*cm² for 638nm excitation and to 4* $\cdot 10^{-16}$*cm² for 785nm excitation. Q was fixed to zero for 638nm excitation and for emission detected below 800nm, while it was individually fitted for all other measurements, with emission detected above 800nm (plotted in inset). The mean of the fitted Q-values for 638nm excitation was 0.36 and 2.4 for 785nm excitation.*

(B): Corresponding experimental and fitted TRAST curves as in (A), recorded in ethanol. In the fitting, σ_N *was fixed to 2.1* $\cdot 10^{-16}$*cm² for 638nm excitation and 6.59* $\cdot 10^{-16}$*cm² for 785nm excitation.* k_{10}^{N} *=1/* τ_f*-*k_{iso}*, where* τ_f *was fixed to 1.09ns (as determined by TCSPC, Table S1).*

k_{iso1}, k^{Th}_{biso1} *and* k^{Th}_{biso2} *were fitted globally for all curves. The fitted curves were found to well reproduce the experimental data, with the following fitted parameter values:* k_{iso}*=6.2μs*$^{-1}$*,* k_{th1} *=0.0042μs*$^{-1}$ *and* k_{th2}*=0.05μs*$^{-1}$*. The cross-sections at 638nm excitation were fitted to* $\sigma_{biso1} = 0.012 \cdot 10^{-16}$*cm*^{2} *and* $\sigma_{iso2} = 0.43 \cdot 10^{-16}$*cm*^{2}*, and were fitted to scale by a factor of F=5 to the corresponding cross-sections at 785nm excitation.* σ_{biso2} *was fitted to* $0.8 \cdot 10^{-16}$*cm*^{2} *for 638nm excitation and to 3.5* $\cdot 10^{-16}$*cm*^{2} *for 785nm excitation. Q was fixed to zero for 638nm excitation and for emission detected below 800nm, while it was individually fitted for all other measurements, with emission detected above 800nm (inset). The mean of the fitted Q-values for 638nm excitation was 0.22 and 2.3 for 785nm excitation.*

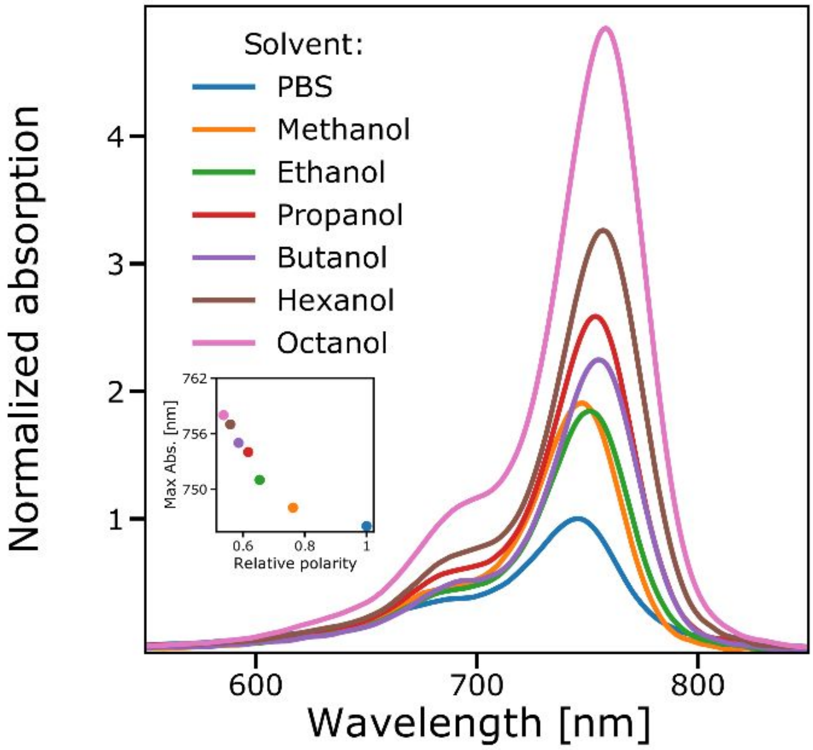

Figure S9. *Absorption spectra of amino-Cy7 in different solvents, normalized with the maximum absorption in PBS. The absorption maximum (Abs_{max}) is shifting to higher wavelengths for higher alcohols, likely coupled to their decreased polarities, as indicated in the legend. Similar to the absorption spectra, the emission maxima were also slightly shifted to longer wavelengths for higher alcohols (data not shown).*

The lifetimes of Cy7A also shows a similar trend as SCy7 and are fitted to values in a similar range. Increasing lifetime with decreased polarity and a second contributing lifetime species is appearing at longer emission wavelengths (data not shown).

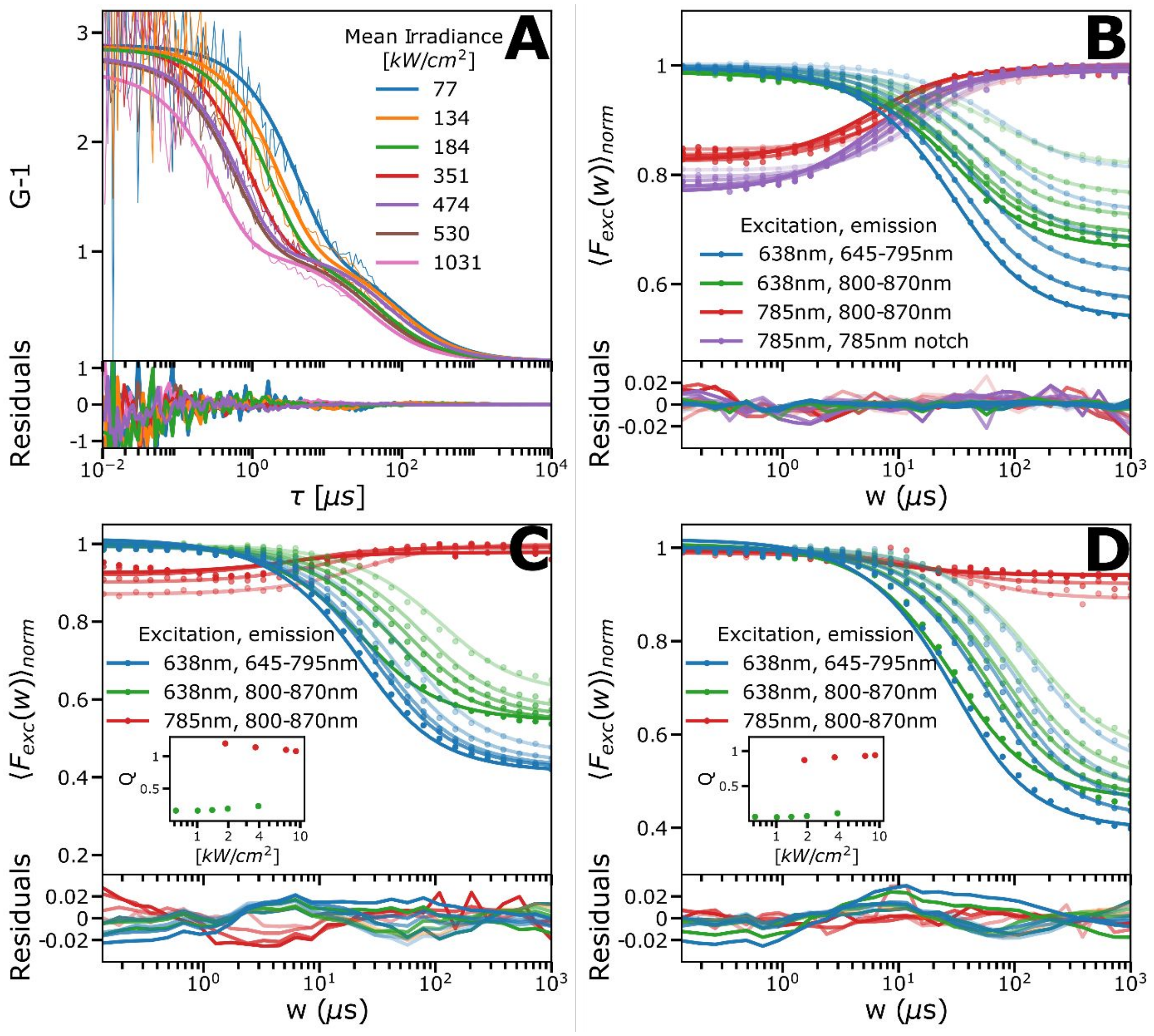

Figure S10.

SCy7 FCS and TRAST data from Figures 1A and 1B (PBS), Figure S8A (methanol) and Figure S8B (ethanol), now fitted by a two-state isomerization model (the model of Figure 3A, without P_2 (and k'_{iso2} and k'_{biso2}), and with P_1 fluorescent with a relative brightness Q). Fitted parameters are given in Table S3.

(A-B): SCy7 (1μM) in PBS (12mM, pH 7.2).

(A): FCS-curves recorded from SCy7 (same as in Figure 1A), 638nm excitation, emission filter: 645-795nm. Fitted curves to the two-state isomerization model (thick lines), fitting residuals below.

(B): TRAST-curves recorded from SCy7 (same as Figure 1B) using a 638nm (blue and green dots) or 780nm (purple and red dots) excitation laser. Fitted curves to the two-state isomerization model (lines) with fitting residuals (below).

(C-D): TRAST curves recorded from SCy7 (1µM) in Methanol (C) and Ethanol (D) using different excitation wavelengths (638nm and 785nm) and emission filters (645-795nm and 800-870nm), as stated in legends (same as in Figures S8A and S8B). Fitted curves to the two-state isomerization model (lines) with fitting residuals (below) Insets show fitted Q-values for the different excitation wavelengths and irradiances applied.

Solvent	τ_f*[ns]*	τ_{f2}*[ns]*	A_{P2} (638nm exc.)	A_{P2} (780nm exc.)
PBS	*0.52*	*0.3*	*0.59*	*0.53*
Methanol	*0.95*	*0.4*	*0.66*	*0.7*
Ethanol	*1.09*	*0.4*	*0.5*	*0.69*
Propanol	*1.16*	*0.4*	*0.4*	*0.63*
Butanol	*1.2*	*0.4*	*0.47*	*0.58*
Hexanol	*1.2*	*0.5*	*0.38*	*0.47*
Octanol	*1.2*	*0.5*	*0.28*	*0.42*
DMSO	*1.4*	-	-	-

Table S1. *Fitted parameter values from TCSPC fluorescence decay measurements of SCy7 in different solvents (Figures S7A-C). As described in Figure S7,* τ_f *was fitted from data obtained at 638nm excitation in the 645-795nm emission range and then kept fixed.* τ_{f2} *and* A_{P2} *were thereafter fitted to the fluorescence decay data obtained at 780nm excitation and with emission detected in the 800-870nm range. For the decay data at 638nm excitation and with emission detected in the 800-870nm range, both lifetimes were fixed, and* A_{P2} *was fitted freely. For the fluorescence decay measurements in PBS, the lifetimes were very short and close to the IRF, and the fits to the bi-exponential model thus more uncertain. Except for PBS, fitted* A_{P2} *values are in general higher at 780nm excitation than at 638nm excitation.*

Φ_{exc} *[kW/cm²]*	A_{P2}
2	*0.38*
4	*0.47*
11	*0.59*
20	*0.62*
30	*0.66*
78	*0.66*

Table S2. *Fitted* A_{P2} *amplitudes from the fluorescence decay data in Figure S7D, versus excitation intensity applied in the TCSPC measurements.*

	FCS, PBS (638nm)	TRAST, PBS (638nm)	TRAST, PBS (785nm)	TRAST, MeOH (638nm)	TRAST, MeOH (785nm)	TRAST, EtOH (638nm)	TRAST, EtOH (785nm)	Unit
k_{iso1}	13.3	12.7	12.7	8	8	5.5	5.5	μs^{-1}
σ_{biso1}	0.006	0.006	15.6 x 0.006	0.001	10x0.001	0.00065	10.8x0.00065	$\cdot 10^{-16} cm^2$
k_{biso1}^{Th}		0.016	0.016	0.0042	0.0042	0.003	0.003	μs^{-1}
Q		0.29 (R-filter)	2.5 (B-filter)<br>3.1 (R-filter)	~0.2 (R-filter)	~1.1 (B-filter)	~0.1 (R-filter)	~0.9 (B-filter)	

Table S3. *Fitted parameters obtained from fitting the curves in Figure S10 by a single-state isomerization model, where P has a brightness Q in relation to N.*
